# Reduced Crossover Interference and Increased ZMM-Independent Recombination in the Absence of Tel1/ATM

**DOI:** 10.1371/journal.pgen.1005478

**Published:** 2015-08-25

**Authors:** Carol M. Anderson, Ashwini Oke, Phoebe Yam, Tangna Zhuge, Jennifer C. Fung

**Affiliations:** Department of Obstetrics, Gynecology, and Reproductive Sciences and Center for Reproductive Sciences, University of California, San Francisco, San Francisco, California, United States of America; National Cancer Intitute, UNITED STATES

## Abstract

Meiotic recombination involves the repair of double-strand break (DSB) precursors as crossovers (COs) or noncrossovers (NCOs). The proper number and distribution of COs is critical for successful chromosome segregation and formation of viable gametes. In budding yeast the majority of COs occurs through a pathway dependent on the ZMM proteins (Zip2-Zip3-Zip4-Spo16, Msh4-Msh5, Mer3), which form foci at CO-committed sites. Here we show that the DNA-damage-response kinase Tel1/ATM limits ZMM-independent recombination. By whole-genome mapping of recombination products, we find that lack of Tel1 results in higher recombination and reduced CO interference. Yet the number of Zip3 foci in *tel1Δ* cells is similar to wild type, and these foci show normal interference. Analysis of recombination in a *tel1Δ zip3Δ* double mutant indicates that COs are less dependent on Zip3 in the absence of Tel1. Together these results reveal that in the absence of Tel1, a significant proportion of COs occurs through a non-ZMM-dependent pathway, contributing to a CO landscape with poor interference. We also see a significant change in the distribution of all detectable recombination products in the absence of Tel1, Sgs1, Zip3, or Msh4, providing evidence for altered DSB distribution. These results support the previous finding that DSB interference depends on Tel1, and further suggest an additional level of DSB interference created through local repression of DSBs around CO-designated sites.

## Author Summary

Meiosis is the type of cell division used by sexually reproducing organisms to create gametes (eggs and sperm, in animals). During meiosis, the two copies of each chromosome swap segments of DNA, forming reciprocal exchanges called crossovers. Crossovers are needed to help ensure that each gamete inherits a copy of every chromosome. Exchange occurs at deliberately induced double-strand DNA breaks, a subset of which become crossovers. In this study we investigate the role of the Tel1/ATM checkpoint kinase in modulating meiotic recombination in budding yeast. We find that in the absence of Tel1, recombination is increased, crossover distribution is altered, and crossovers are less dependent on the Zip3 protein, which mediates the major crossover pathway in yeast. We also find evidence which we infer indicates that Tel1, the helicase Sgs1, and the crossover-promoting proteins Zip3 and Msh4 influence how breaks are positioned throughout the genome. These results are consistent with a role for Tel1 in regulating the spacing of breaks along chromosomes. Our results also suggest that crossover-committed sites may suppress break formation in surrounding areas. Such a feedback mechanism would allow cells to achieve a sufficient number of crossovers without sustaining excess DNA breaks, which are inherently risky.

## Introduction

Sexual reproduction depends on meiosis, a specialized type of cell division that produces haploid gametes from diploid cells. Recombination between homologous chromosomes is a key feature of the first meiotic division. A subset of recombination events creates reciprocal exchanges known as crossovers (COs), which help ensure that homologs segregate properly in meiosis I. Recombination also includes non-reciprocal events called noncrossovers (NCOs). The number and distribution of COs are highly regulated to ensure proper chromosome segregation. A striking feature of the CO landscape is the non-random spacing of COs, a phenomenon known as interference (reviewed in [1]). As a result of interference, COs tend to be relatively evenly spaced along chromosomes. Although interference was first reported over a century ago as the decreased probability that a CO would occur if another CO occurred nearby [2], its mechanistic underpinnings are still not well understood.

**Fig 1:**
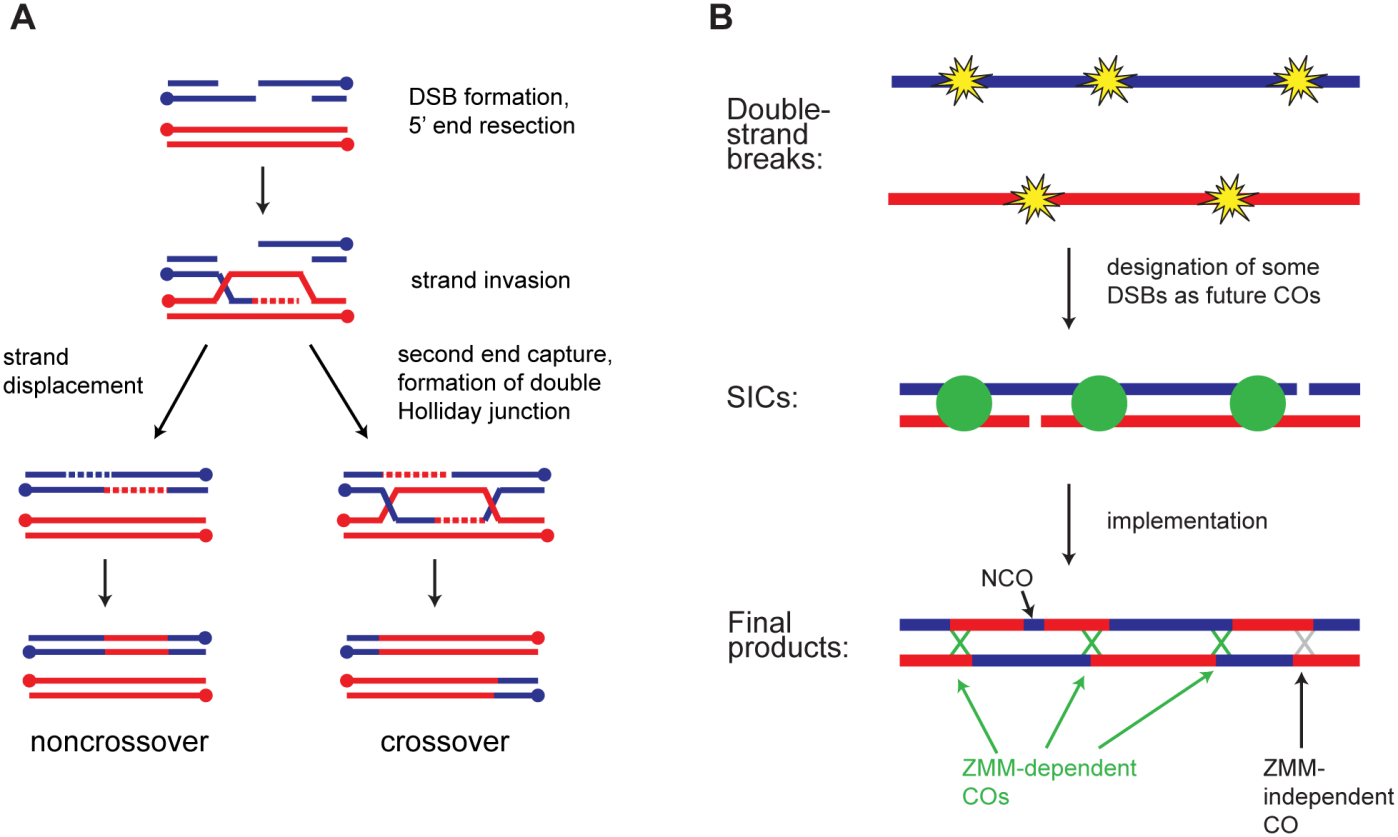
Overview of meiotic recombination. A) Major recombination pathways. A Spo11-induced DSB is resected to expose single-stranded tails. A 3’ tail invades a homologous duplex and is extended using the homolog as a template. Displacement of the invading strand leads to NCO formation by synthesis-dependent strand annealing (SDSA). Alternatively, capture of the second DSB end leads to formation of a dHJ. In wild type, dHJs are typically resolved as COs, but NCO formation is also possible. B) CO patterning. During or soon after DSB formation, a subset of DSBs becomes committed to the CO fate. These sites are marked by SICs and show interference. Subsequent steps convert CO-committed sites into COs. The majority of non-SIC-marked sites become NCOs, but some of them may also become COs.

Both COs and NCOs arise from double-strand DNA breaks (DSBs) induced by the Spo11 enzyme [3]. How each DSB’s fate is determined is poorly understood, but several findings indicate that a decision is made prior to formation of stable strand invasion intermediates [4,5,6]. Formation of both COs and NCOs begins with resection of DSBs to expose 3’ single-stranded tails that can invade homologous duplex DNA (Fig 1A). At sites of future COs, initial strand invasion is followed by formation of stable intermediates known as single-end invasions and double Holliday junctions (dHJs) [4,6]. Normal timing and levels of these CO-specific intermediates require the ZMM proteins (Zip2-Zip3-Zip4-Spo16, Msh4-Msh5, Mer3) [5]. Upon pachytene exit, dHJ-containing intermediates are resolved to form COs. In contrast, NCOs appear prior to pachytene exit, without formation of stable intermediates, and without the need for ZMMs [4,5,6]. Thus COs and NCOs show distinct timing, intermediates, and genetic dependencies, but how the repair pathway is initially chosen at each DSB is unknown.

In budding yeast, a subset of COs is associated with cytologically observed foci known as synapsis-initiation complexes (SICs) [7,8]. SICs contain the ZMM proteins and appear to promote polymerization of the synaptonemal complex (SC). Multiple lines of evidence indicate that SICs form at CO-committed sites. [9,10,11,12]. SICs, like COs, show interference [9,13,14,15,16]. Strikingly, however, in certain deletion mutants the distribution of SICs (cytological interference) is normal even though CO interference as assessed genetically is defective (e.g. *zip1Δ*, *msh4Δ*, and *sgs1Δ*) [9]. Based on these findings a two-phase model for establishment of CO interference has been proposed (Fig 1B) [5,9]. First, DSBs are formed and designated as future sites of COs or NCOs, with SICs marking CO-committed sites. Second, these sites are processed into their respective products. According to this model *zip1Δ*, *msh4Δ*, and *sgs1Δ* cause defects in the implementation phase without disrupting the initial CO/NCO decision. SICs thus provide a readout of repair pathway choice.

Formation of SICs requires the presence of Spo11-induced DSBs [8,10]. SICs are seen in the processing-defective *rad50S* strain, in the recombination-defective *dmc1Δ* strain, and in haploid cells, indicating that normal DSB processing and interhomolog recombination are not required for SIC formation [7,8,17,18], thus prompting us to ask whether recombination pathway choice hinges on events immediately after break induction.

In mitotic cells, where the response to DSBs has been extensively characterized, the earliest known events after DSB formation are the binding and activation of proteins involved in the DNA damage response, including Mre11-Rad50-Xrs2 (MRX), Tel1, Mec1, and the 9-1-1 complex (Ddc1-Mec3-Rad17 in budding yeast) [19]. MRX and Tel1 are recruited to unresected DSBs, while Mec1 and 9-1-1 respond to single-stranded DNA (ssDNA). Since SICs are seen in the processing-defective *rad50S* mutant, we reasoned that Tel1, which responds to unprocessed DSBs, might play a role in SIC formation.

Tel1/ATM is known to control meiotic DSB levels. In mice, loss of ATM causes a dramatic increase in DSB frequency [20]. In flies, mutation of the ATM ortholog *tefu* causes an increase in foci of phosphorylated H2AV, suggesting an increase in meiotic DSBs [21]. Measurements of DSB frequency in *tel1Δ* yeast have given conflicting results, with three studies showing an increase [22,23,24] and two showing a decrease [25,26]. All but one of these studies relied on mutations that prevent DSB repair (*rad50S* or *sae2Δ*) to enhance detection of DSBs. These mutations may themselves influence the number and distribution of DSBs, confounding interpretation of the results. The one study that examined DSB levels in *tel1Δ* single mutants found a convincing increase in DSBs [23].

Tel1/ATM also influences the outcome of recombination. In mice, loss of ATM causes meiotic arrest due to unrepaired DSBs [27,28,29]. Infertility due to a failure to produce mature gametes is a feature of the human disease ataxia telangiectasia, suggesting that ATM is also required for meiotic DSB repair in humans. Meiotic progression in *Atm* ^*−/−*^ mice can be partially rescued by heterozygosity for *Spo11* [30,31]. Compared to *Spo11* ^*+/−*^ alone, *Spo11* ^*+/−*^ *Atm−* ^*/−*^ spermatocytes show synapsis defects and higher levels of MLH1 foci, a cytological marker for COs [30]. In these spermatocytes the spacing of MLH1 foci is less regular and the sex chromosomes often fail to form a CO in spite of greater overall CO frequency. These results point to a role for ATM in regulating the distribution of COs. In yeast, examination of recombination intermediates at the *HIS4LEU2* hotspot found that Tel1 is required for efficient resection of DSBs when the overall number of DSBs genome wide is low [32]. Under these conditions, the preference for using the homolog as a repair template was decreased in the absence of Tel1.

Tel1 also regulates DSB distribution (reviewed in [33]). In budding yeast DSBs are distributed non-uniformly throughout the genome, falling into large “hot” and “cold” domains spanning tens of kb, as well as smaller hotspots of a few hundred bp or less [3]. DSBs, like COs, are thought to show interference. Direct measurement of DSBs at closely spaced hotspots found that the frequency of double cuts on the same chromatid was lower than expected under a random distribution [23]. These calculations could only be done in repair-defective mutants due to detection issues, but nevertheless provide the most compelling evidence to date of DSB interference. This study found that DSB interference in yeast depends on *TEL1*. The existence of DSB interference was originally proposed based on the observation that introduction of a new hotspot greatly reduces DSB frequency in nearby areas [34,35,36,37]. It remains unknown whether this hotspot-hotspot competition and DSB interference represent the same phenomenon. A careful examination of recombination products at the *HIS4LEU2* hotspot found evidence that DSBs also inhibit each other in *trans*, i.e. between chromatids, and that *trans* inhibition depends on Tel1 [24]. The authors proposed that spreading of *trans* inhibition along chromosomes could contribute to even spacing of DSBs.

Several proteins with key meiotic roles are subject to Tel1/Mec1-dependent phosphorylation, although in many cases the individual contribution of Tel1 (separate from Mec1) has not been tested. These include the axial protein Hop1, the Spo11 accessory factor Rec114, histone H2A, Sae2, and Zip1 [22,38,39,40]. Tel1-dependent phosphorylation of Rec114 may at least partially account for Tel1 regulation of DSB levels, although this has yet to be definitively tested [22]. Loss of Tel1 causes only a mild defect in spore viability and little or no delay in meiotic progression [39,41].

Multiple lines of evidence indicate that interactions between homologs influence DSB formation (reviewed in [42]). Experiments in worms first led to the proposal that nascent COs inhibit additional DSBs on the same chromosome [43,44]. This mechanism would allow DSB formation to continue until each chromosome has achieved a CO. Studies of worms, mice, and yeast indicate that some aspect of homolog engagement, possibly SC formation, leads to inhibition of DSBs [45,46,47,48]. High-resolution mapping of DSBs in synapsis-defective yeast found a change in the genome-wide distribution of DSBs in populations of cells [47]. To our knowledge, no previous studies have assessed whether regular spacing of DSBs along individual chromosomes is dependent on synapsis or other interhomolog interactions.

Our lab and others have developed techniques for mapping recombination products genome-wide in budding yeast [49,50,51,52]. We mate two yeast strains, S96 and YJM789, with sequence differences at about 65,000 sites. After recovery of the four progeny of a single meiosis, we use next-generation sequencing or microarrays to genotype progeny. The resulting map allows us to deduce the locations of all COs and nearly all NCOs with a median resolution of 81 bp.

Using this technique, we show here that loss of Tel1 causes an increase in recombination along with decreases in CO interference and the CO/NCO ratio. Yet the number of SICs in *tel1Δ* cells is similar to wild type, and these SICs show normal interference. These results suggest that in the absence of *tel1Δ*, a substantial number of COs arises from a ZMM-independent pathway. Our analysis of recombination in *tel1Δ zip3Δ* confirms this conclusion. Furthermore, we also see a change in the distribution of all recombination products in *tel1Δ*, *zip3Δ*, *msh4Δ* and *sgs1Δ*, which we infer indicates a change in DSB distribution. Since SIC distribution is normal in these strains (except *zip3Δ*, which lacks SICs) this result implies that DSB interference is not required for proper patterning of CO precursors. We argue that the opposite is true: the CO patterning process contributes to DSB interference, as CO-designated sites repress formation of additional DSBs in surrounding areas.

## Results

### Loss of Tel1 increases recombination and alters its outcome

**Fig 2:**
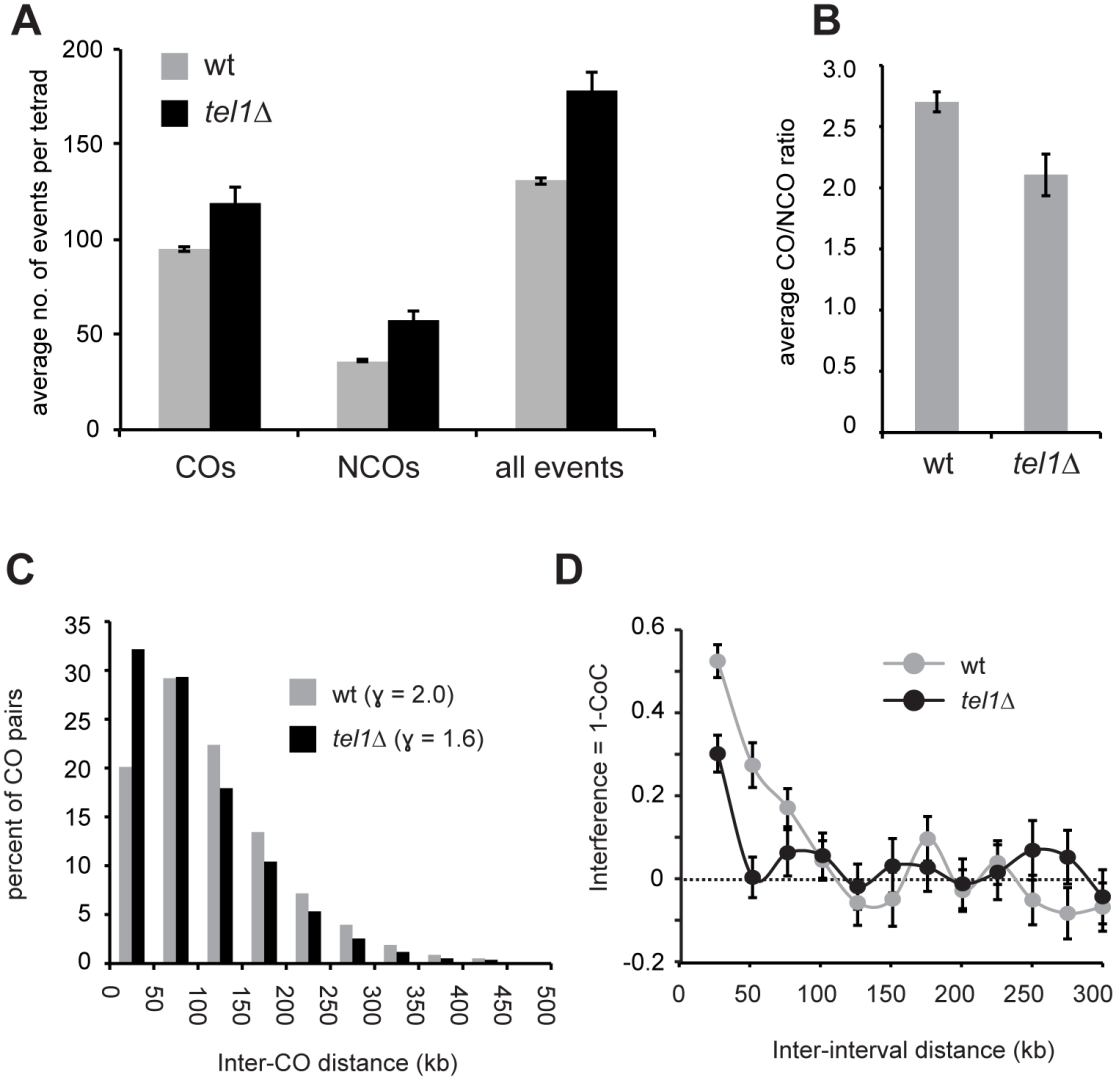
Absence of Tel1 alters the outcome of recombination. A) The average number of COs, NCOs, and all events (COs + NCOs) per tetrad is shown. COs include event types E2, E3, E5, E6, and E7 as defined in [53] and Fig 3. NCOs include E1 and E4. B) The average ratio of COs to NCOs is shown for wt and *tel1Δ*. C) Histogram of distances between pairs of adjacent COs. D) Interference (1 –CoC) for COs in wild-type and *tel1Δ* tetrads. For each inter-interval distance, the CoC was calculated individually for all possible interval pairs genome-wide, and the average is plotted. For all plots, analysis of COs used data from 52 wild-type and 14 *tel1Δ* tetrads; analysis of NCOs and all events used data from 52 wild-type and eight *tel1Δ* tetrads. Error bars: standard error (SE).

**Fig 3:**
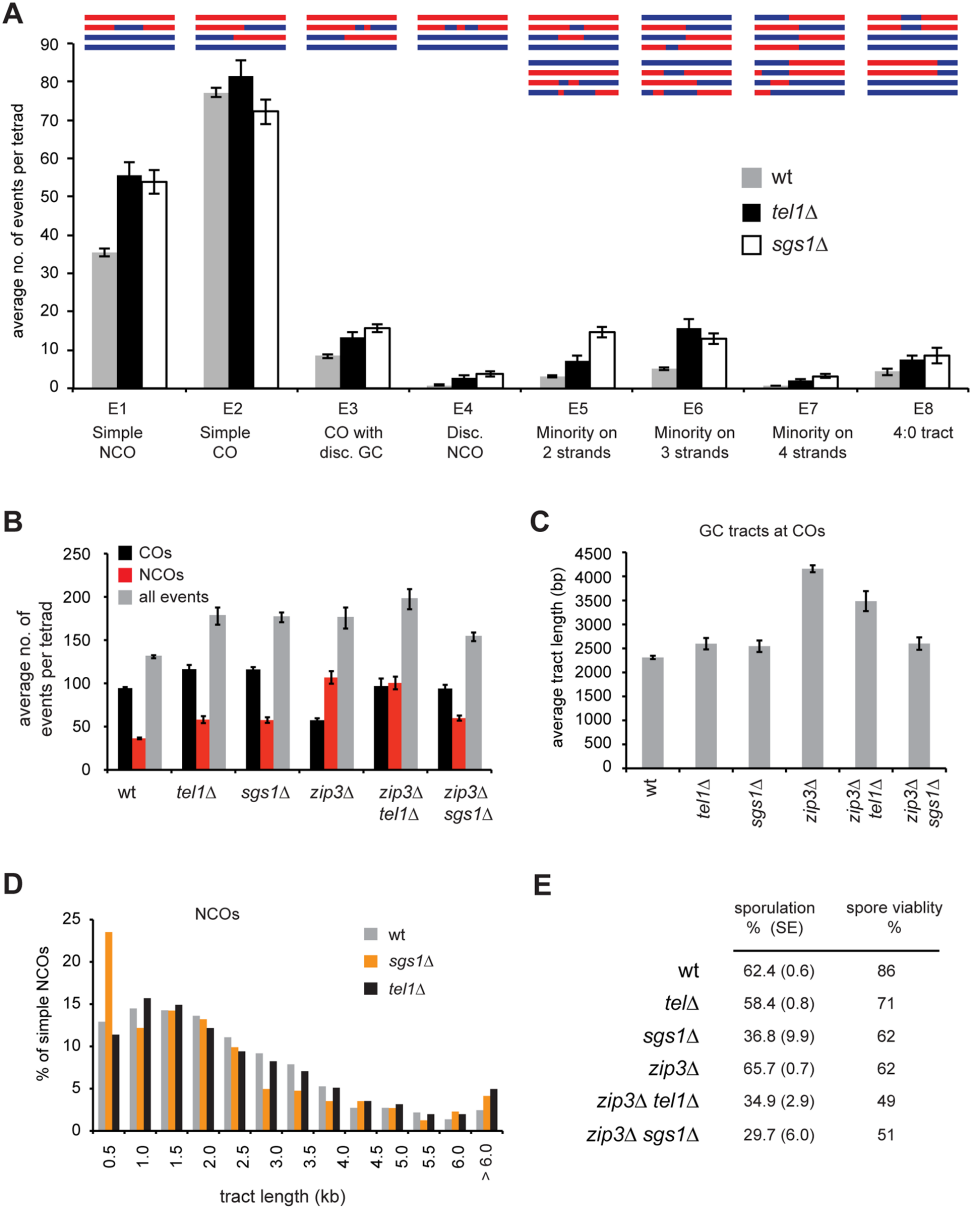
*tel1Δ* and *sgs1Δ* show distinct recombination phenotypes. A) The average number of each product type is shown. Event types are as defined in [53]. “Disc” = discontinuous. B) The average number of COs, NCOs, and all events is shown. COs include E2, E3, E5, E6, and E7. NCOs include E1 and E4. Plots of all contributing event types are in S2 Fig. C) The average length of GC tracts at simple COs (E2) is shown. D) Histogram of the lengths of simple NCOs (E1). Error bars in all plots: SE. For all plots except analysis of COs in part B, data were derived from 52 wild-type, eight *tel1Δ*, nine *sgs1Δ*, seven *zip3Δ*, six *zip3Δ tel1Δ*, and six *zip3Δ sgs1Δ* tetrads. Analysis of CO frequency in part B used an additional set of six *tel1Δ*, four *sgs1Δ*, and 23 *zip3Δ* tetrads genotyped at lower resolution. Calculations of E8s in wild type used only the six wild type tetrads sequenced in our lab (see Materials and Methods). E) Sporulation frequency was measured in three independent cultures of each genotype, with the exception of *sgs1Δ* for which only two cultures were used. At least 300 cells per culture were counted. Average and SE are shown. The distribution of spores per ascus is shown in S3D Fig. Viability was measured for at least 200 tetrads per genotype.

To investigate the role of Tel1 in meiotic recombination, we identified recombination products genome-wide in the progeny of 14 *tel1Δ* hybrid diploids. Eight tetrads were genotyped at high resolution by next-generation sequencing and used for analysis of both NCOs and COs, while six were genotyped at lower resolution and used for analysis of COs only. As wild-type controls, we used data from 46 tetrads genotyped by high-density tiling array [51] and six wild-type tetrads sequenced in our lab [53]. As expected based on analysis of recombination at a single hotspot [24], deletion of *TEL1* significantly increases the overall rate of recombination (Fig 2A). This finding is also consistent with reports that DSB levels are increased in *tel1Δ* [22,23,24], although our data should be taken only as a rough estimate of DSB levels, since not all DSBs produce detectable products. In addition, there is potential for selection bias in our results since we are only able to assay cells that complete meiosis and produce viable spores. In the case of *tel1Δ* this bias is expected to be mild since the defects in sporulation and spore viability are quite modest (Fig 3E). We find that NCOs are increased disproportionately: the mean number of NCOs per tetrad increases by 60%, while COs increase by only 23%, resulting in a significantly lower CO/NCO ratio in *tel1Δ* compared to wild type (Fig 2B; *p* = 0.009; Student’s t-test).

The lower CO/NCO ratio in *tel1Δ* suggests that loss of Tel1 alters repair pathway choice. Another readout of pathway choice is CO interference, which refers to the relatively even spacing of COs in wild type. One way to assess interference is to analyze distances between adjacent COs (Fig 2C). In wild-type cells, inter-CO distances are well fit by a gamma distribution [50]. The value of the shape parameter γ of the best-fit distribution indicates the strength of interference, with γ > 1 indicating positive interference and γ = 1 indicating random distribution. γ is reduced from 2.0 in wild type to 1.6 in *tel1Δ* (Fig 2C), revealing a decrease in interference. Since γ is sensitive to changes in CO density, we also analyzed interference using the coefficient of coincidence (CoC) method in which the frequency of COs in two intervals is compared with the expected frequency of double COs under an assumption of no interference. Interference expressed as 1 –CoC also shows a significant decrease in *tel1Δ* (Fig 2D; *p* < 0.0001, chi-square test).

### Higher frequency of complex products in *tel1Δ*

*tel1Δ* cells show a striking increase in complex products containing discontinuous gene conversion (GC) tracts or genotype changes on multiple chromatids (Fig 3A). To classify recombination products, we merge changes within 5 kb of each other into a single “event” that is assigned to one of eight event types (E1-E8). Except for E8, all of the types were previously defined [53]. 5 kb was chosen based on prior analysis of wild-type tetrads showing that events within 5 kb have distinct properties suggesting they arise from a single DSB [49]. All figures use a 5 kb threshold for merging unless otherwise specified. Results without merging are qualitatively similar and are shown in S1, S2B, S3, S6 and S8A Figs. In our classification system, “simple NCOs” (E1) and “simple COs” (E2) are products without any other genotype switches within 5 kb. A “CO with discontinuous GC” (E3) is a CO with a nearby GC tract (within 5 kb). A “discontinuous NCO” (E4) contains two GC tracts within 5 kb of each other. We also identify three categories of “minority” events (E5-E7), which are ambiguous products that could arise more than one way. For example, a minority event on three chromatids (E6) could be two closely spaced COs or a CO with a nearby NCO. In the current study we add a new category, E8, containing 4:0 tracts. These may represent cases of two overlapping NCOs, or may arise from pre-meiotic recombination. In wild type, complex events (categories E3-E7) account for about 14% of all meiotic recombination products; in *tel1Δ* they represent 22% of products, a statistically significant difference (*p* < 0.0001, Student’s t test). We see a similar increase in complex events in *sgs1Δ* ([53] and Fig 3A). The phenotypes of *tel1Δ* and *sgs1Δ* show several other similarities. Both mutants have higher recombination frequency, a decrease in the CO/NCO ratio, and a moderate decrease in CO interference [53,54,55,56]. These similarities suggested that Tel1 and Sgs1 might act together in regulating recombination.

### Sgs1 and Tel1 have distinct roles in regulating recombination

Sgs1 is thought to control recombination pathway choice by unwinding nascent strand invasion intermediates unless they are protected by ZMMs [54]. Deletion of *SGS1* rescues CO levels in *zmm* mutants [54,55]. We find that *tel1Δ* and *sgs1Δ* rescue crossing over in *zip3Δ* to similar extents (Fig 3B). However, in other ways *tel1Δ* and *sgs1Δ* show dramatically different phenotypes. First, loss of Zip3 causes a striking increase in NCOs. This increase is largely suppressed by *sgs1Δ* but not by *tel1Δ* (Fig 3B). Second, *zip3Δ* displays abnormally long GC tracts associated with COs (Fig 3C and [53]). This tract lengthening is suppressed by *sgs1Δ* [53] but only partially suppressed by *tel1Δ*. Third, a notable feature of recombination in *sgs1Δ* is the presence of a population of very short NCOs that we propose arise from aberrant SDSA [53]. This cohort of short NCOs is not seen in *tel1Δ* (Fig 3D). Together these results indicate that Sgs1 and Tel1 have distinct roles in regulating recombination.

### SIC abundance and interference are similar in wild type and *tel1Δ*

**Fig 4:**
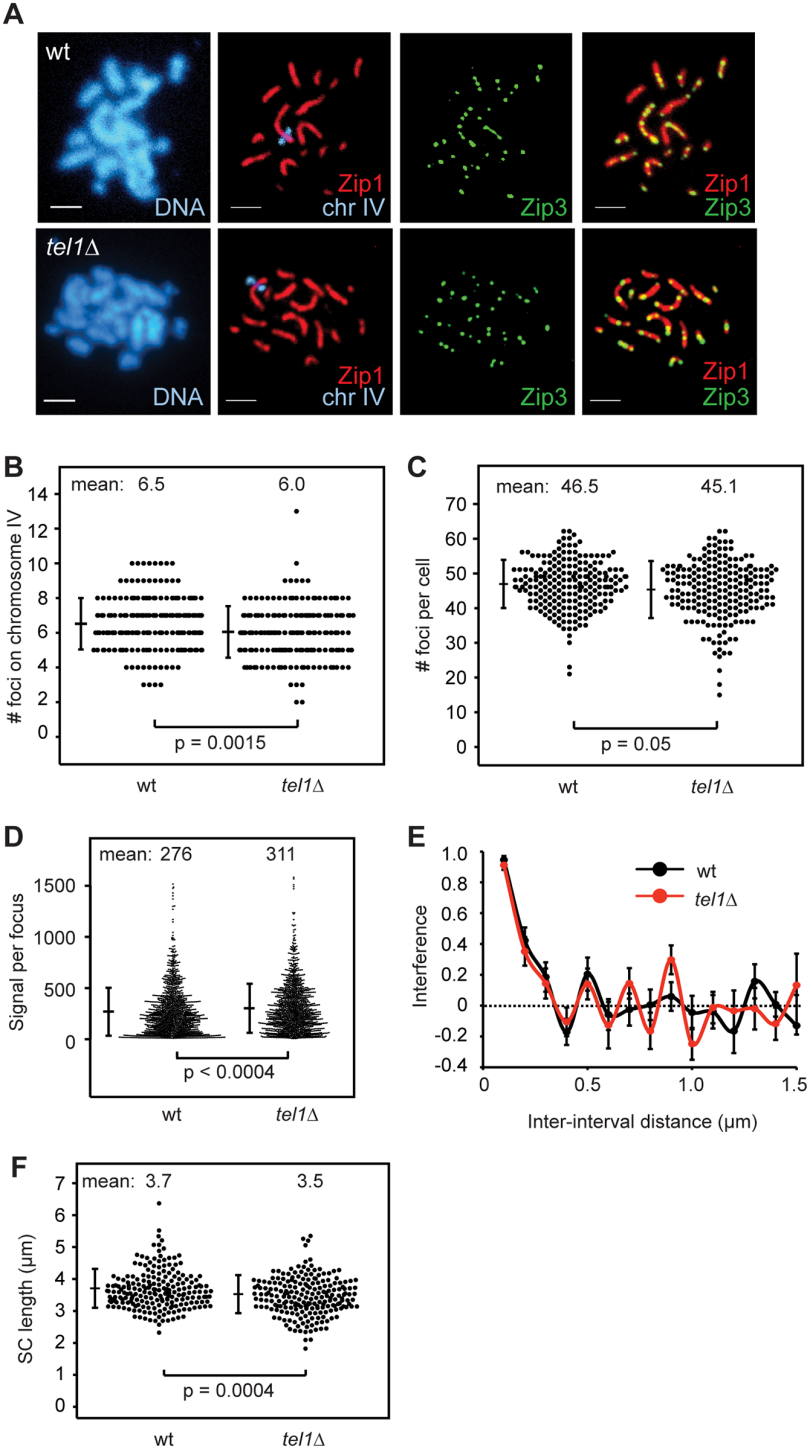
SIC abundance and interference in *tel1Δ* are similar to wild type. A) Meiotic chromosomes from wild type and *tel1Δ* were spread and labeled with antibodies to Zip1 (red) and Zip3-GFP (green). An array of tet operators on the right arm of chromosome IV was identified by coexpression of a tetR-mCherry fusion; the native mCherry signal was used for visualization. Scale bar: 2 μm. B) The number of Zip3-GFP foci on chromosome IV in 204 wild-type and 202 *tel1Δ* nuclei with full synapsis. Data are pooled from five independent experiments using two independent isolates of each strain. Small but significant decreases in the average number of foci on chromosome IV (7%), and in the total number of foci per cell (3%) were observed for *tel1Δ*. Individual experiments are shown in S5 Fig. Significance is lost if the most striking experiment is removed. Bars: mean and standard deviation (SD). C) Number of Zip3 foci per cell determined by automated focus finding in ImageJ, using the same images scored in B. Three of five contributing experiments showed a decrease in Zip3 foci, and the difference is statistically significant in two of the three; the other two experiments showed a slight increase in *tel1Δ*, one of which is statistically significant. Bars: mean and SD. D) Intensity of Zip3 foci. Wild-type and *tel1Δ* cells marked with arrays of tet operators on either chromosome IV or XIV were mixed on the same slide. The genotype of each cell was identified based on the size of the labeled chromosome. Four different slides were analyzed with equal numbers of wild-type and *tel1Δ* images from each slide (25 cells of each genotype total). Both labeling schemes (wild type marked on chromosome XIV and *tel1Δ* marked on chromosome IV, and vice-versa) gave similar results; figure includes data from both sets of strains. Total intensity of each focus is plotted. E) Interference calculated as 1-CoC for Zip3-GFP foci on chromosome IV from 72 wild-type and 76 *tel1Δ* cells. Error bars: SE. F) Chromosome IV Zip1-stained SC lengths in 209 wild-type and 212 *tel1Δ* nuclei. Data are pooled from five independent experiments using two independent isolates of each strain; each of the five individual experiments shows a decrease in SC length in *tel1Δ*, and the difference is statistically significant in two of the five. Individual experiments are shown in S5 Fig. Bars: mean and SD.

To determine whether Tel1 acts upstream or downstream of SIC formation we measured the number and positions of Zip3 foci on chromosome IV or on all chromosomes in pachytene spreads of wild type and *tel1Δ* (Fig 4A). We find that *tel1Δ* cells show no increase in Zip3 foci compared to wild type in spite of greater numbers of COs and DSBs (Fig 4B and 4C). Since the number of foci in *tel1Δ* could be underestimated if foci are less intense and thus more difficult to detect, we determined whether the intensity of foci is similar in wild type and *tel1Δ*. By mixing both strains on a single slide, we control for slide-to-slide variation in staining. The two strains were labeled with arrays of tet operators on chromosomes of dramatically different size, allowing the genotype of individual cells to be identified after imaging. We find that the intensity of Zip3 foci in *tel1Δ* is slightly higher than in wild type (Fig 4D), indicating that the lack of increase in focus abundance is not caused by detection problems. Detection of Zip3 foci could also be impaired if foci are closer together in *tel1Δ*, causing adjacent foci to appear as a single merged focus. However, we find that the median distance between pairs of adjacent foci is similar in the two strains (0.42 μm in wild type vs. 0.44 μm in *tel1Δ*, a difference that is not statistically significant (S4A Fig)). We would also expect an increase in focus size if many more adjacent foci were unresolvable in *tel1*. This is not the case since the size of individual foci is the same in the two strains (S4B Fig). Together these results indicate that *tel1Δ* does not cause an increase in Zip3 foci. Zip3 foci in *tel1Δ* also show normal interference as determined by CoC analysis (Fig 4E).

SC length has been shown to correlate with the number of cytologically distinguishable CO-committed sites in worms and mammals [57,58] and not necessarily with the total CO number [59,60,61]. We find that the mean length of chromosome IV SC is 6% shorter in *tel1Δ* than in wild type (Fig 4F; p = 0.0004, Student’s t test). Thus in yeast, SC length parallels the number of SICs and not the overall number of COs.

**Fig 5:**
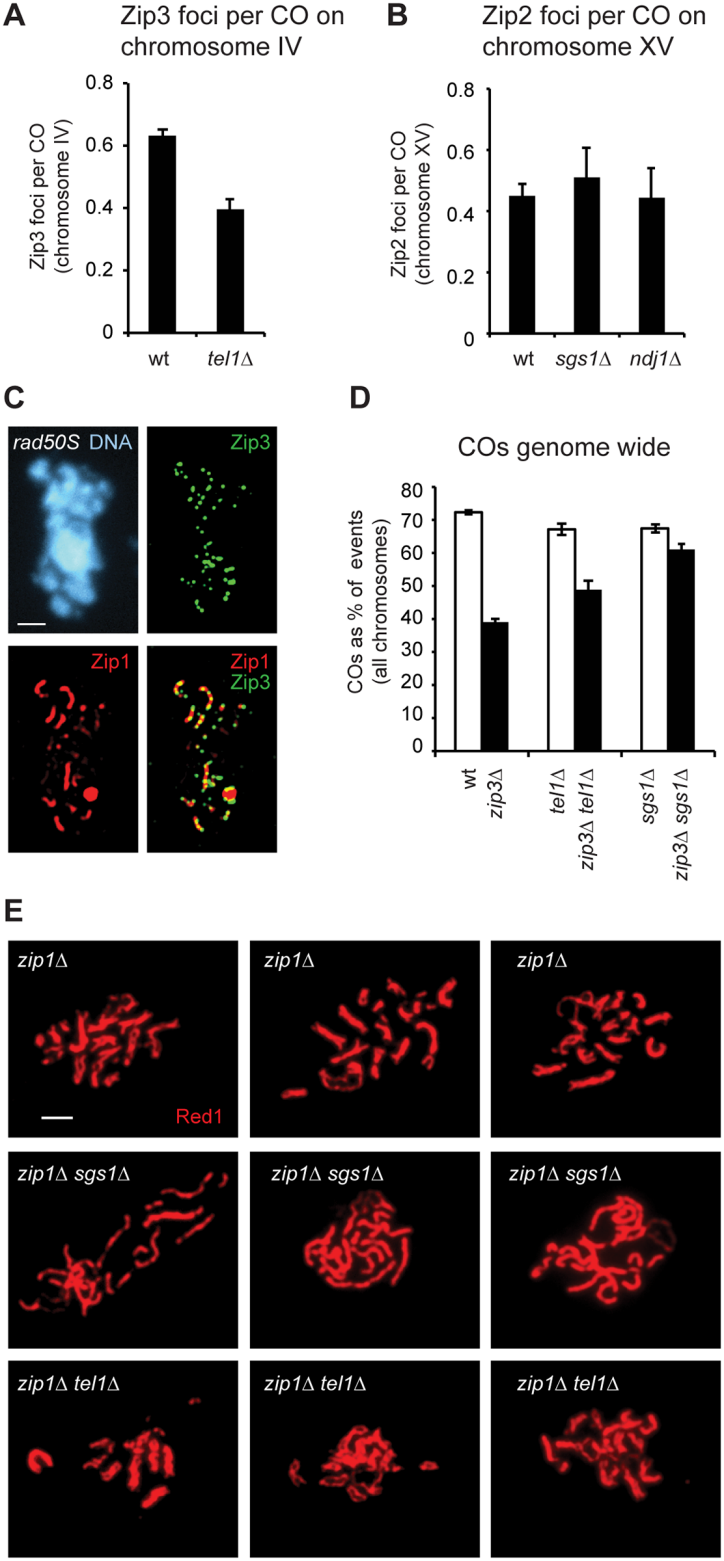
COs are less Zip3 dependent in *tel1Δ*. A) The average number of Zip3-GFP foci on chromosome IV detected on spreads (as in Fig 4) divided by the average number of COs on chromosome IV in genotyped tetrads (as in Fig 2A). B) The average number of Zip2 foci on chromosome XV detected on spreads [9] divided by the average number of COs on chromosome XV in genotyped tetrads (this study and [50].) C) Meiotic chromosomes from *rad50S* cells prepared as in Fig 4A. D) The average number of COs genome wide expressed as a percent of all interhomolog events. Per-tetrad averages are shown. E) Pachytene spreads stained with anti-Red1 antibody to detect axes. Three examples are shown for each genotype. Error bars: SE.

The lack of increase in SIC abundance in *tel1Δ* is unexpected because three previously tested mutants with higher levels of COs (*sgs1Δ*, *pch2Δ*, and *ndj1*) had more SICs, while mutants with fewer COs (*msh4Δ* and *zip1Δ*) had fewer SICs [9,17]. By comparing the number of COs on chromosome IV in our recombination mapping experiments with the number of Zip3 foci on chromosome IV, we calculated a ratio of SICs to COs (Fig 5A). This ratio should be viewed as a rough estimate, since the measurements of SICs and COs were performed in different strains (isogenic and hybrid diploids, respectively). In wild type, the SIC/CO ratio is 0.63, implying that the majority of COs occur at SICs. In *tel1Δ* this ratio is reduced to 0.40, suggesting that non-SIC-associated COs are the major class. For comparison we determined the SIC/CO ratio in two other mutants with increased CO levels, *sgs1Δ* and *ndj1Δ*. For this analysis we compared the number of Zip2 foci on chromosome XV with the number of COs on that chromosome, both from published studies [9,50]. We find no significant change in the SIC/CO ratio in these mutants compared to wild type (Fig 5B). These results reveal a specific role for Tel1 in regulating the fraction of SIC-associated COs.

We considered the possibility that the failure of *tel1Δ* cells to make more Zip3 foci than wild type might be caused by DSB processing defects. A role for Tel1 in resection of meiotic DSBs has been suggested [32,39,62] Yet high levels of Zip3 foci are seen in the resection-defective *rad50S* strain (Fig 5C and [7]). These results indicate that resected ends are not required for formation of SICs.

### A larger share of COs in *tel1Δ* is ZMM-independent

Non-ZMM associated COs, often called Class II COs, are assumed to lack interference [63,64,65]. A possible reason for decreased CO interference in *tel1Δ* is that non-ZMM-associated COs, which represent a minority of events in wild-type cells, make up a larger share of events in *tel1Δ*. To further test this we compared the effect of deleting *ZIP3* on CO abundance in wild type and *tel1Δ* (Fig 5D). To adjust for different DSB frequencies, we normalized CO numbers by expressing them as a percent of all interhomolog events. The percent of events resolved as COs drops from 72% in wild type to 39% in *zip3Δ*. As predicted, the decrease in COs between *tel1Δ* (67%) and *tel1Δ zip3Δ* (49%) is more modest. Thus COs in *tel1Δ* show less ZMM dependence than in wild type. An even more dramatic decrease in ZMM dependence is seen in *sgs1Δ*: CO frequency is similar in *sgs1Δ* (67%) and *sgs1Δ zip3Δ* (61%). We conclude that in *tel1Δ*, SICs are still at least partially functional in terms of promoting the CO fate, since loss of Zip3 in *tel1Δ* causes a decrease in COs. The opposite is true in *sgs1Δ*: SICs are either not fully functional or not functionally relevant in terms of promoting COs, since very little effect was seen upon deleting *ZIP3*.

### *tel1Δ* does not cause pseudosynapsis in *zip1Δ*

In cells lacking the SC central element Zip1, synapsis is lost and axes are held together at a few sites per chromosome, termed axial associations. The exact nature of these links is unknown, but they are thought to correspond to SIC-marked sites [8]. In the *zip1Δ sgs1Δ* double mutant, axes are held closely together by a dramatic increase in the number of axial associations, a phenomenon referred to as pseudosynapsis [56]. Given the similar numbers of recombination products in *tel1Δ* and *sgs1Δ* (Fig 3A), we tested whether pseudosynapsis also occurs in *zip1Δ tel1Δ*. We find strikingly distinct phenotypes in *zip1Δ sgs1Δ* and *zip1Δ tel1Δ* (Fig 5E). In *zip1Δ sgs1Δ*, virtually no regions of axial separation are seen, whereas many sites of axis separation are visible in *zip1Δ tel1Δ*, similar to *zip1Δ* alone. This is consistent with the finding that SICs are increased in *sgs1Δ* but not in *tel1Δ*, and supports the idea that axial associations occur at SICs. Alternatively, the close association of axes in *zip1Δ sgs1Δ* may arise from aberrant structures, such as trapped recombination intermediates, found only in *zip1Δ sgs1Δ* and not in *zip1Δ tel1Δ*.

### Analysis of all detectable recombination products suggests that DSB interference depends on Tel1, ZMMs, and Sgs1

To test whether Tel1 mediates DSB interference we examined the distribution of all recombination products in our *tel1Δ* tetrads, using all interhomolog events as a proxy for DSBs. A potential concern relating to this analysis is that we are unable to detect some recombination events. These include intersister events, estimated to arise from 15–30% of all DSBs [66], and NCOs falling between markers or in which mismatch repair restored the original genotype, together estimated to include 30% of interhomolog NCOs [51]. However, failure to detect a percentage of the DSB population per se should not affect the calculated strength of interference since CoC does not vary significantly with event density [15], a fact that we verified by randomly removing events from a wild-type data set to simulate loss of detection (S7 Fig). The inability to detect some events would only be problematic if the undetected events were distributed non-uniformly throughout the genome. Previous analysis of the genome-wide distribution of COs and NCOs found good agreement between recombination frequencies in wild type and DSB frequencies in *dmc1Δ* [51], indicating that the distribution of detectable interhomolog events reflects the underlying DSB distribution.

**Fig 6:**
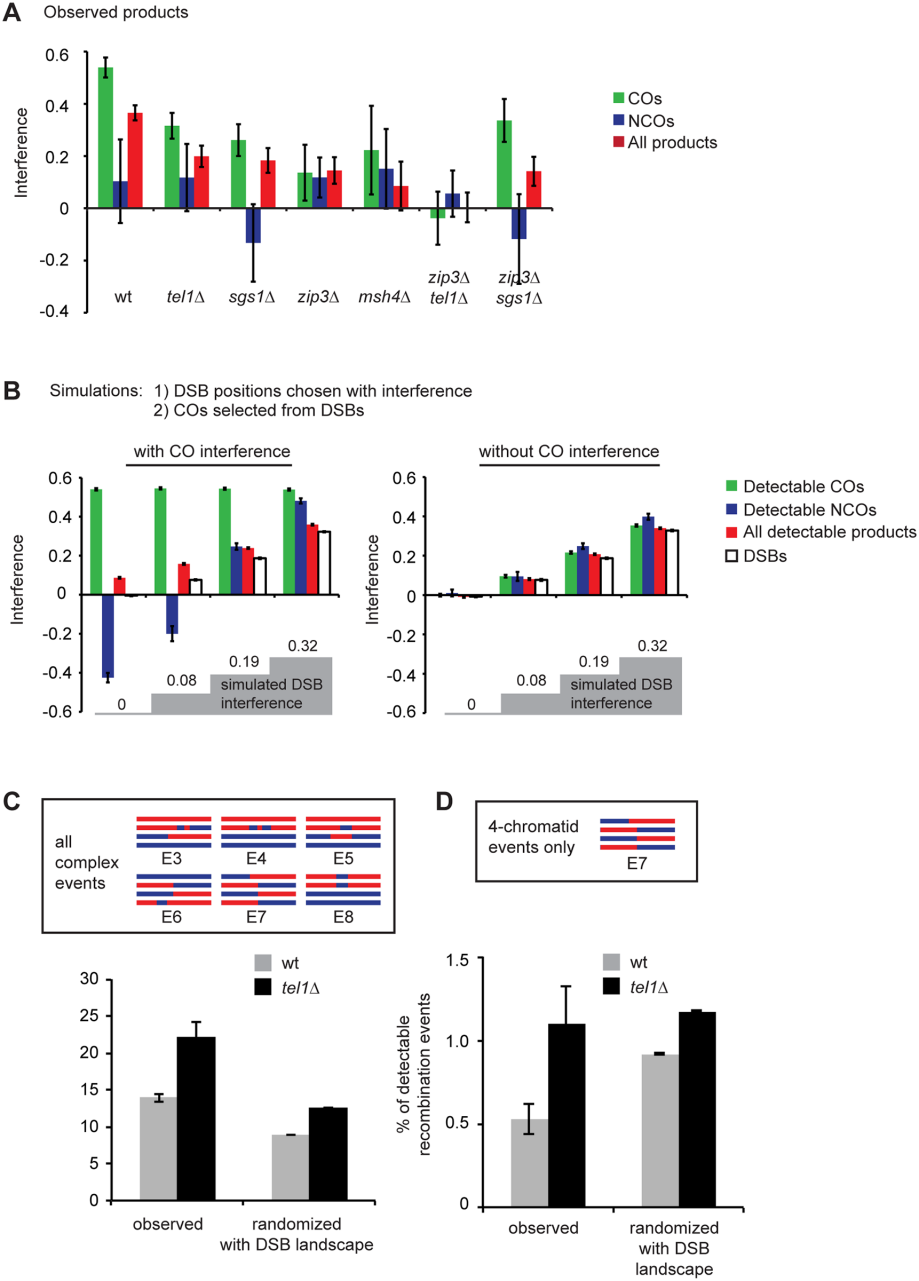
The distribution of recombination events is altered in *tel1Δ*, *sgs1Δ*, and *zmmΔ*. A) Interference calculated as 1-CoC for a bin size and inter-interval distance of 25 kb is shown for COs only, NCOs only, or all events from whole-genome recombination data. *msh4Δ* data comprise seven tetrads sequenced in our lab and five tetrads genotyped by Mancera et al. [51]. B) Simulations were performed in which an interfering population of DSBs was first created, and then COs were selected from the DSBs. COs were selected either with or without additional interference. Remaining DSBs were considered NCOs. Failure to detect some events was simulated by removing 20% of all events and 30% of the remaining NCOs. Interference between all simulated DSBs or between “detectable” products is shown. Left: the strength of DSB interference was varied, and the strength of CO interference was selected to recapitulate observed interference between COs in wild type. Right: conditions were the same as on the left except no CO interference was incorporated. C) “Complex” events include the event types shown, and are events that could arise from more than one DSB. Randomized data consist of at least 10000 simulated tetrads per genotype in which the CO and GC tract positions in real tetrads were randomized. “With DSB landscape” indicates that event positions take into account DSB frequencies (see Materials and Methods). D) As in C, but includes only events involving four chromatids. Error bars: SE.

We find that the distribution of all interhomolog events in wild type displays interference, and this interference is decreased (from 0.37 to 0.21) in *tel1Δ* (Fig 6A; *p* = 0.0007; chi-square test). We infer that Tel1 mediates DSB interference, in agreement with physical assays [23].

Unexpectedly, we find that the combination of all interhomolog products in *zip3Δ*, *msh4Δ*, and *sgs1Δ* also shows reduced interference (from 0.37 in wild type to 0.14, 0.11, and 0.21, respectively; *p* = 0.0003, 0.004, and 0.002 respectively). These results suggest that DSB interference is defective in these mutants. These three mutants are known to disrupt CO interference, but to our knowledge they have not been proposed to affect DSB-DSB spacing. Based on these results, we hypothesize that CO designation and/or formation of a SIC suppresses formation of DSBs nearby. Several previous studies point towards the existence of feedback between interhomolog interactions and DSB formation [43,44,45,46,47,48] and indicate that there is considerable temporal overlap between DSB and SIC formation [47,67,68]. We suggest that, beyond controlling the levels of DSBs, some aspect of CO designation also shapes the pattern of DSBs along individual chromosomes.

One potential question in interpreting these results is whether reduced interference among COs would automatically be expected to cause reduced interference among all detectable products, even without an underlying change in DSB interference. To test this we performed a simulation in which DSB interference was established entirely independently of CO interference. All DSB positions were first selected (with interference), and then CO positions were selected (with additional interference) from the DSBs, with the remaining DSBs becoming NCOs. We then randomly removed 20% of all events to simulate intersister repair, and 30% of the remaining NCOs to simulate loss of detection due to restoration and lack of markers. Results are shown for a wild-type level of CO interference with various levels of DSB interference (Fig 6B, left), and for the same conditions without CO interference (Fig 6B, right). These simulations illustrate several points. First, in the presence of CO interference, the strength of interference between all detectable recombination products is slightly higher than the true DSB interference among all four chromatids. This is due to preferential detection of COs (i.e., we detect essentially all COs, which strongly interfere, but we fail to detect some NCOs, which do not). Second, the level of interference between NCOs varies with the strength of DSB and CO interference. At low levels of DSB interference, selection of strongly interfering COs from an almost randomly spaced pool of DSBs results in NCOs that show negative interference, i.e. a tendency to cluster. At high levels of DSB interference, imposition of CO interference enhances the regular spacing of both COs and NCOs. In this model, to achieve a level of interference between all products equivalent to what is observed in wild type, it is necessary to impose strong DSB interference (1-CoC = 0.32). At this level of DSB interference, NCOs show strong interference. In contrast, NCOs in wild type do not show significant interference (Fig 6A). In wild type, interference for NCOs alone is 0.1, which does not differ significantly from no interference (*p* = 0.18). In addition, there are no statistically significant differences between wild type and any of the mutants in the strength of interference between NCOs. This lack of interference among NCOs lends support to the notion that DSB interference is at least partially driven by DSB suppression near COs. If DSB interference arose entirely independently of COs, we would expect NCOs to show interference.

Third, these simulations show that complete loss of CO interference only slightly reduces the interference among all detectable events (Fig 6B, compare left and right panels). This reduction is too small to account for the observed reductions in *tel1Δ*, *zip3Δ*, *msh4Δ*, and *sgs1Δ*.

It should be noted that in these simulations, DSB interference was applied to all four chromatids equally; i.e., a DSB on one chromatid suppressed DSBs equally along the same chromatid and along the three other chromatids, a situation that might not occur in vivo. We have separately simulated situations where DSB interference exclusively affects DSBs on the same chromatid or on the same pair of sister chromatids (S8B Fig). We found that it was not possible to recapitulate the observed strength of DSB interference among all four chromatids when the simulated DSB interference only affected DSBs on the same chromatid. Simulations in which DSB interference acted on a chromatid and its sister were capable of recapitulating the wild-type level of interference among all events on all chromatids, but this simulation again predicted much stronger interference among NCOs than is actually observed. In reality, DSB interference may arise from a combination of same-chromatid, intersister, and interhomolog effects, but our simulations suggest that none of these scenarios can account for the observation of very weak interference among NCOs if we assume DSB interference is entirely independent of CO designation. These results do not rule out that DSB interference may be partially created upstream of CO designation, but they suggest that such a mechanism does not solely account for the observed distribution of events.

### Multi-chromatid recombination products in *tel1Δ* likely result from decreased DSB interference along with increased DSB frequency

A previous study of the *HIS4LEU2* hotspot found many tetrads with multiple COs and/or GC tracts in both wild type and *tel1Δ* (20% and 36% of detectable recombination products, respectively) interpreted as arising from multiple DSBs [24]. To test whether the complex recombination events we observed in *tel1Δ* could be caused by closely spaced DSBs, we modeled a total loss of DSB interference by randomizing the positions of COs and GC tracts in our unmerged *tel1Δ* or wild-type data. GC tracts falling within the boundaries of a CO were not randomized since they are assumed to arise from the same DSB as the CO.

In the simulation, we incorporated the DSB landscape, such that the probability of an event falling in a particular area was determined by the frequency of DSBs in that region [69]. We then merged genotype changes within 5 kb into a single event and classified them as event types E1-E8. Zhang et al. [24] classified recombination products as T0, T1, or T2 based on the inferred number of initiating DSBs. We consider our event types E3-E8 as equivalent to T2 events (inferred to arise from two DSBs). Some of these event types could not be detected by Zhang et al. due to the limited number of markers available at *HIS4LEU2*. Surprisingly, we find that events inferred to arise from two DSBs occur more frequently in wild type than expected based on random chance (Fig 6C). If a specific mechanism existed to prevent these events, we would expect the opposite: these events should be more frequent in randomized data than in real tetrads. The high number of these events may reflect the fact that such events could arise from a single DSB; for example, three-chromatid events could result from two ends of a DSB invading different chromatids. Such multi-chromatid events were proposed to underlie the high number of complex products potentially arising from two DSBs in the *sgs1-ΔC795* mutant [24]. Alternatively, DSBs in both wild type and *tel1Δ* might show negative interference, i.e. a tendency to cluster. If so, this effect would presumably operate only over short distances (less than 5 kb), since we see positive interference when genotype changes within 5 kb are treated as a single event (Fig 6A). In accordance with this, concerted formation of DSBs on the same chromatid within an approximately 8 kb range was observed in *tel1Δ* cells by a physical assay [23].

Due to the ambiguous origins of two- and three-chromatid events, we separately analyzed four-chromatid events (E7). We consider these more likely to be cases of more than one DSB occurring in *trans* (i.e. on different chromatids), since only a very aberrant recombination event could produce genotype switches on all four chromatids from a single DSB. We find that the frequency of four-chromatid events in wild type is significantly lower than the frequency expected due to random chance (Fig 6D; *p* = 0.0007; Student’s t test). In contrast, the frequency of these events in *tel1Δ* is statistically indistinguishable from the frequency expected due to random chance (Fig 6D; *p* = 0.78) These results support the conclusions of Zhang et al. that a Tel1-dependent mechanism suppresses the occurrence of more than one DSB per quartet of chromatids. As noted by Zhang et al. and Garcia et al. [23,24], *trans* inhibition could operate either between sister chromatids or between homologs. Our analysis of E7 products cannot distinguish between these two models, since we are unable to determine whether the initiating DSBs occurred on homologs or sisters. In theory, E8 products (4:0 tracts), which are increased in *tel1Δ*, may represent cases where DSBs occurred on both sisters. However, such products can also arise from premeiotic gene conversions. We find that the majority of E8 events have perfectly overlapping endpoints (i.e., gene conversion tracts beginning and ending at the same markers on both chromatids). Of the 4:0 tracts that are not part of a complex event, 72% (in wild type) or 74% (in *tel1Δ*) have perfect overlap. Such a high degree of overlap would not be expected if the majority of these events represented independent NCOs. Therefore we suspect that the *tel1Δ-*dependent increase in these events may arise from an increase in premeiotic recombination. Some, but not all, previous studies of recombination in vegetatively growing *tel1Δ* cells have found an increase [70,71,72].

Our simulations show that complex products arising from multiple DSBs are expected to occur more often in hot genome regions compared to cold regions (S8C and S8D Fig). This trend may explain the unusually high number of complex events seen by Zhang et al. at *HIS4LEU2*, an artificial hotspot with higher DSB frequency than natural hotspots.

## Discussion

### Tel1 is involved in an early step in recombination pathway choice

**Fig 7:**
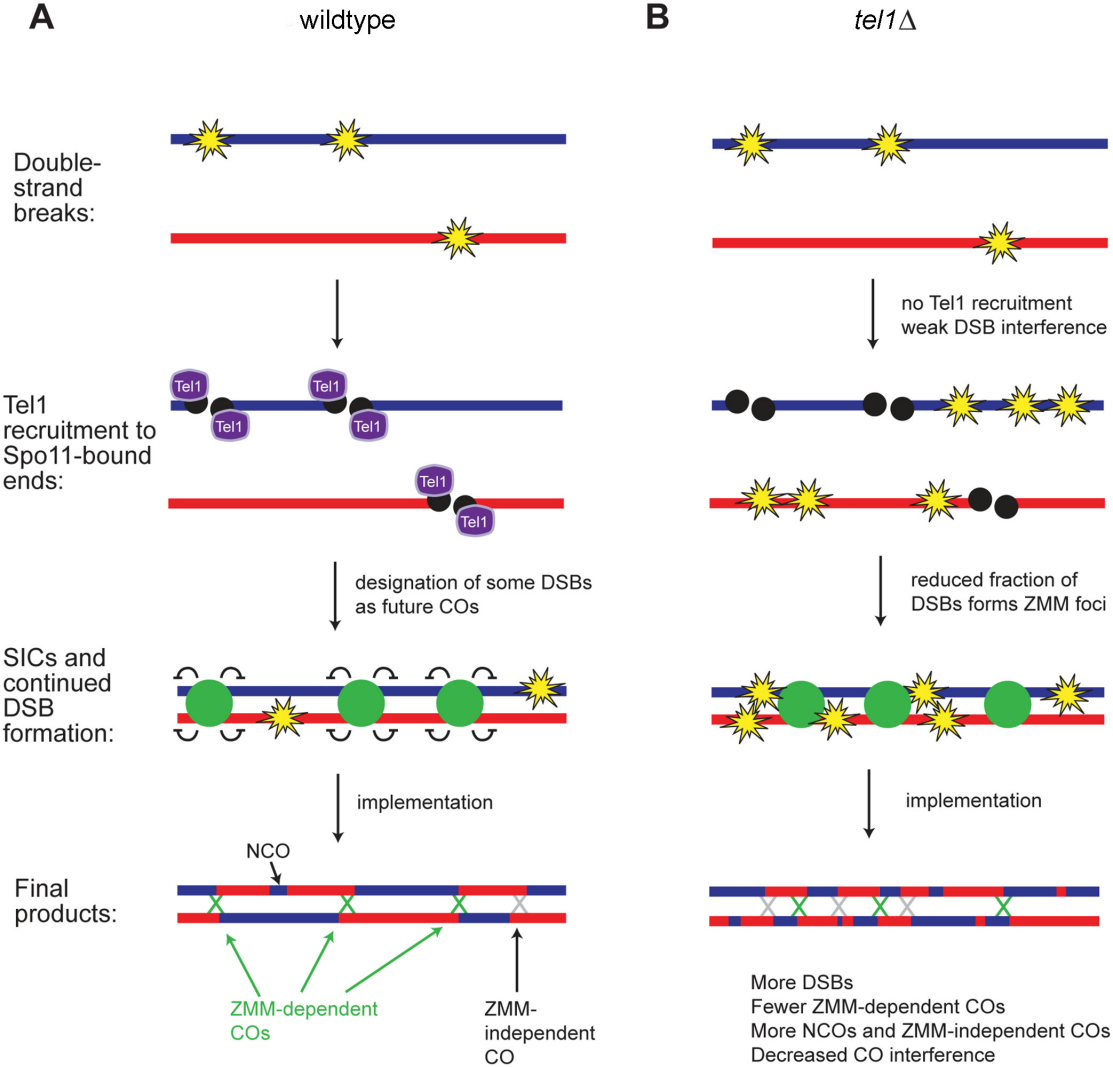
Model for recombination pathway choice with and without Tel1. A) In contrast to Fig 1 where DSB formation and CO designation were depicted as independent processes, we propose that formation of a SIC suppresses DSB formation nearby, so that later DSBs tend not to occur near a SIC. Early forming DSBs thus have a greater tendency to become interference-capable CO-designated sites and later DSBs tend to become NCOs or “non-interfering” COs. B) In *tel1Δ*, the number of DSBs is higher than in wild type and DSB distribution is less regular. A smaller fraction of DSBs becomes committed to the CO fate and marked by SICs; SICs still show an orderly distribution, as in wild type. DSBs not marked by SICs become NCOs or “non-interfering” COs, leading to decreased CO interference.

Our data indicate that Tel1 is required for an early step in recombination pathway choice (Fig 7). In the absence of Tel1, the ratio of COs to NCOs, CO interference, and the dependence of COs on *ZIP3* are all decreased, indicating that a greater proportion of recombination events occurs via non-ZMM-dependent mechanisms. The abundance of SICs is also similar to wild type, which is surprising given the higher levels of DSBs and COs in *tel1Δ*. Zhang et al [16] found modestly increased numbers of SICs in *tel1Δ* in the SK1 strain background, (11% increase on chromosome XV). Given the differences in strain backgrounds and chromosomes analyzed, these may represent essentially the same result. In SK1, the increase in SICs was smaller than the increase in DSBs (50% increase at *HIS4LEU2* in *a rad50S* background) and COs (23% increase at *HIS4LEU2*) previously reported in SK1 [24]. Thus both studies point to the conclusion that the number of SICs per CO is reduced in *tel1Δ*.

Taken together, our results suggest two non-mutually-exclusive mechanisms for the modulation of recombination by Tel1. One possibility is that in *tel1Δ* there are two distinct populations of DSBs: a normal cohort of DSBs repaired as in wild type, and a population of “excess” DSBs repaired via non-ZMM-dependent pathway(s). Another model consistent with our results is that *tel1Δ* causes a general defect in commitment of DSBs to the ZMM-dependent CO pathway. The wild-type-like number of foci in *tel1Δ* may be the net result of a decrease in SIC-forming ability partially offset by an increase in the abundance of DSBs. If Tel1 does promote SIC formation, other factors must have functional overlap with Tel1 in this role, since SICs show normal abundance in *tel1Δ*. We speculate that Tel1 phosphorylation of ZMMs may promote their recruitment to specific DSBs. All of the ZMM proteins contain multiple SQ/TQ sites, the consensus sequence for Tel1/Mec1 phosphorylation. Mutation of the four SQ/TQ sites in Zip3 reduces its association with DSB hotspots and reduces CO frequency in some intervals, suggesting its ability to form a SIC is impaired [11]. However, *zip3-4AQ* causes only a mild decrease in COs and no loss of spore viability, indicating that other relevant Tel1 targets in addition to Zip3 must exist.

Our results confirm that interference among CO-committed sites is not defective in *tel1Δ*, as previously reported [16]; instead, poor CO interference arises from the fact that many COs in *tel1Δ* occur via a non-ZMM pathway. Our analysis of recombination outcomes in *tel1Δ zip3Δ* provides experimental evidence for the prediction that in mutants with higher levels of DSBs without an increase in SICs, “extra” DSBs would be channeled into ZMM-independent repair pathways [15].

In previous observations of *Atm*−/− *Spo11 +/−* mouse spermatocytes [30], MLH1 served as a cytological marker for CO positions. Loss of ATM caused a decrease in interference between MLH1 foci, whereas Zip3 foci in yeast show normal interference (this study and [16]). MLH1 foci are often assumed to mark all COs rather than only ZMM-associated COs [73], although this view is not universally accepted (for example, [74,75,76].) If the view that MLH1 foci mark all COs is correct, the decreased interference between MLH1 foci would be consistent with our genome-wide mapping of *tel1Δ* recombination products, which showed decreased overall CO interference. Alternatively, ATM may play distinct roles in CO patterning in mammals and yeast.

COs are often categorized as Class I (ZMM-dependent) or Class II (Mus81-Mms4 dependent), with only Class I COs participating fully in CO interference. In *tel1Δ* the additional non-ZMM COs may be typical Class II COs dependent on Mus81-Mms4, or may form by another mechanism. Regardless of the mechanism, due to not participating in ZMM-dependent CO patterning, they would be expected to show decreased CO interference. Class II COs are often described as “non-interfering”, but as noted by Zhang et al. this terminology is probably inaccurate [16]. Since all sites of recombination are influenced by DSB interference, even Class II COs are expected to show weak interference.

### Evidence for Tel1-mediated DSB interference

The distribution of all events in *tel1Δ* is consistent with a decrease in interference between DSBs. Effects of *tel1Δ* on DSB spacing have been previously reported [23,24], but it was not necessarily obvious that this would be detectable at the level of all recombination products genome wide. Garcia et al. observed a defect in DSB interference along single chromatids, but could not assay interference among all four chromatids in a homolog pair [23]. The genetic analysis by Zhang et al. observed *trans* inhibition among all four chromatids at a particular hotspot, but could not determine whether such inhibition extends laterally along chromosomes [24]. It is thus striking that a defect in interference among all recombination products is detectable in our data among all four chromatids and at distances of tens of kb. This supports the proposal of crosstalk between homologs in determining DSB positions [24].

### Crossover designation may regulate DSB positioning

The distribution of all events in *zip3Δ* and *msh4Δ* also implies a decrease in interference between DSBs. The inferred decrease in DSB interference in *zip3Δ* and *msh4Δ* suggests that CO designation and/or formation of a SIC suppresses formation of DSBs nearby (Fig 7a). Consistent with this model, recent analysis of the genome-wide DSB distribution in a population of *zip3Δ* cells found that regions with the greatest change in DSB frequency in *zip3Δ* were enriched for Zip3 binding in wild type [47]. This strongly suggests that the influence of Zip3 on DSBs is at least partially a local effect, and is not solely attributable to chromosome-wide or nucleus-wide effects such as altering the timing of synapsis. Importantly, this model explains why CO-NCO pairs show interference while NCO-NCO pairs do not [51]. One implication of this model is that earlier-forming DSBs would have a greater tendency to become CO-designated sites compared to later-forming DSBs. In support of this, Zip3 localization is reduced at hotspots believed to represent late-forming DSBs [11]. A prediction of the model is that any mutation causing changes in SIC distribution or defects in SIC formation will also cause changes in DSB distribution. This may explain a recent observation in *hed1Δ dmc1Δ* cells, which have a reduced number of SICs. In this mutant CO distribution is altered such that the difference in recombination rates between adjacent hot and cold regions is diminished [18]. This was interpreted as indicating a change in the distribution of DSBs, with cold regions sustaining more DSBs as a result of delayed pairing or synapsis. We suggest that decreased SIC formation may also contribute to this change in DSB distribution.

The defective DSB interference inferred to occur in *sgs1Δ* may also be mechanistically related to SICs. In the absence of Sgs1, SICs form but appear to be uncoupled from sites of COs. This conclusion is based on the fact that SICs in *sgs1Δ* show normal interference while COs do not (Fig 6A and [9,55]) and that loss of ZMMs in *sgs1* mutants does not significantly diminish CO frequency (Fig 3B and [53,54,55,56]). We speculate that the CO-promoting function of SICs and their putative DSB interference function are both impaired by lack of Sgs1.

How might a CO-designated site suppress nearby DSBs? Several studies have proposed that SC formation, which proceeds from SICs, inhibits DSBs [45,46,47,48]. Axial proteins including the Spo11 accessory complex Rec114-Mei4-Mer2 and HORMAD proteins are excluded from synapsed regions, suggesting mechanisms by which synapsed chromosomes could become refractory to DSB formation [22,48]. Alternatively, an inhibitory signal other than synapsis, such as modification of axial proteins, might spread from CO-designated sites. We note that in yeast, the presence of a homolog is not strictly required for SIC formation [8]. This leaves open the possibility that ZMMs may influence the DSB landscape through mechanisms not involving interactions between homologs. Regardless of the exact molecular nature of the signaling events, such a mechanism would allow cells to create a sufficient number of COs to promote proper chromosome segregation without sustaining excess DSBs, which are inherently risky.

A key question raised by these results is whether Tel1 and ZMMs influence DSB distribution via distinct mechanisms. In our data, the inferred level of DSB interference in *tel1Δ zip3Δ* double mutants is lower than in either single mutant, implying action through different pathways, but the difference is not statistically significant, possibly due to the small size of the data sets. Another observation that suggests Tel1 and ZMMs control DSBs through different mechanisms is their behavior in *sae2Δ* or *dmc1Δ* backgrounds: the *tel1Δ*-dependent increase in DSBs persists in *sae2Δ* or *dmc1Δ*, while *zmm*-dependent increases do not [5,23,47]. However, the aforementioned ZMM experiments measured only DSB levels and not DSB interference [47], which may represent distinct phenomena. One piece of evidence that is difficult to reconcile with Tel1 controlling DSB interference independently of SICs is the fact that NCOs alone do not show a significant level of interference (Fig 6A). This suggests that if SIC-independent DSB interference exists, it is weak, at least when DSBs on all four chromatids are considered. However, some aspect of DSB interference may act only along a particular chromatid or pair of sisters, and such an effect might operate independently of SICs; this effect would be very difficult to detect in our data.

### SIC interference does not require evenly spaced DSB precursors

In spite of low inferred DSB interference, normal SIC interference is seen in *tel1Δ*, *msh4Δ*, and *sgs1Δ* [9]. This result implies that proper patterning of SICs does not require an orderly array of DSBs, and further suggests that DSB interference might not contribute significantly to CO interference in wild type. In *tel1Δ*, poor DSB interference apparently contributes to poor CO interference because many COs occur at non-SIC-marked sites. However, in wild type it is still unclear whether DSB interference plays a role in CO interference.

### Loss of Tel1 decreases *trans* DSB inhibition

Previous studies indicated that wild-type cells limit the occurrence of DSBs on multiple chromatids at a particular hotspot and argued that Tel1 mediates this *trans* inhibition [23,24]. Whether such *trans* inhibition operates between homologs, sisters, or both has been controversial. Zhang et al. argued that *trans* inhibition most likely represented inhibition between homologs, whereas Garcia et al. suggested the opposite, based partly on re-analysis of Zhang et al.’s data. Our analysis of recombination products containing genotype switches on all four chromatids supports the existence of a mechanism limiting multiple DSBs per four chromatids. Since we are unable to determine which chromatids sustained the initiating DSBs, we cannot distinguish whether this one-per-quartet constraint arises from *trans* inhibition between homologs, between sisters, or both.

Our simulations of DSB distributions along chromosomes indicate that multi-DSB events are expected to be more frequent in hot regions compared to cold ones. As a corollary, changes in the frequency of multiple DSBs observed at *HIS4LEU2* or any other locus in mutant strains may reflect a change in the relative hotness of the hotspot or a change in the overall DSB landscape, rather than loss of a specific regulatory mechanism limiting re-cutting. In light of this, experiments involving one or a few hotspots should be interpreted with caution, especially if performed in *rad50S* or *sae2Δ* strains in which DSBs are restricted to a more limited number of hotspots than in wild type [77].

## Materials and Methods

### Yeast strains

Strain genotypes are listed in S1 Table. For recombination mapping, diploids were made by mating S96 and YJM789 haploids. All chromosome spreads were in the BR1919-19B background. Strain construction is described in Supporting Materials and Methods.

### Whole-genome recombination mapping

DNA was prepared for Illumina sequencing using a NextFlex kit (BIOO) with Illumina-compatible indices or as described [49] with 4-base or 8-base inline barcodes. Samples were sequenced in 50-base single-end runs on an Illumina Genome Analyzer or Illumina HiSeq 2000 or 2500 at the Vincent J. Coates Genomic Sequencing Laboratory (UC Berkeley) or the Center for Advanced Technology (UCSF). Genotype determination was performed essentially as described using the ReCombine package [49], but no insertions/deletions were genotyped. Briefly, after genotyping, CrossOver v6.3 was used to detect recombination products without merging close genotype switches. Products within 5 kb were then merged into a single event and sorted into one of seven categories as described [53], but with the addition of the new E8 category containing 4:0 tracts. Only the six wild-type tetrads sequenced in our lab were used to calculate the number of E8 products, since the number of E8s per tetrad was significantly different in the 46 wild-type tetrads genotyped by Mancera et al. Other event types did not show such differences. E8s were not used in any subsequent calculations, including calculations of “total events”, since we consider them likely to arise prior to meiosis.

Raw sequence data have been deposited in the NIH Sequence Read Archive under accession number SRP044001. Data for wild type, *sgs1Δ*, *zip3Δ*, *msh4Δ*, and four out of six *sgs1Δ zip3Δ* tetrads were previously deposited under accession numbers SRP028549 (wild type) and SRP041214 (all other strains). Additional processed data is deposited in Dryad Digital Repository (doi:10.5061/dryad.bj042).

### Meiotic chromosome spreads

Chromosome spreads were made as described [78]. Wild-type, *tel1Δ*, and *rad50S* cells were collected after 15–21 hours in 2% potassium acetate at 30°C. *zip1Δ*, *zip1Δ sgs1Δ*, and *zip1Δ tel1Δ* cells were collected after 19–21 hours. Antibody staining is described in Supporting Materials and Methods. Images were collected on a DeltaVision microscope (Applied Precision). SC lengths and Zip3 focus positions were measured using the 3D model module in Softworx (Applied Precision). To measure focus intensities, foci were found via the Threshold and Watershed functions in ImageJ. The total signal in each focus was measured by the AnalyzeParticles function in ImageJ.

### Interference analysis

Gamma distributions were fitted to inter-event distances [50]. For calculations of CoC, the genome was divided into 25 kb bins. The frequency of events in each bin was calculated, as well as the frequency with which any two pairs of bins on the same chromosome both contained events in the same tetrad. The expected frequency of such double events under a model of no interference is the product of the individual event frequencies in the two bins. The CoC for each pair of bins is the ratio of the observed frequency of double events to the expected frequency. This ratio was calculated for all bin pairs with a non-zero expected frequency, and results were averaged for all bin pairs separated by a given distance. In Fig 6 and S8 Fig, only the results for adjacent bin pairs are plotted. A chi-square test was used to compare expected and observed double COs. Measurements of cytological interference were performed essentially as above, but chromosome IV was divided into 0.1 μm bins.

### Modeling DSB and CO interference

For simulations in Fig 6B, a Python script was used to generate 1000 simulated tetrads for each set of conditions. The genome was divided into bins of 100 bp, and the number of DSBs in each tetrad was chosen from a normal distribution based on observed event frequencies in wild type. DSB positions were sequentially chosen, and a gamma hazard function was used to reduce the probability of DSBs in nearby bins after each DSB position was selected. After selection of DSB positions, CO positions were chosen by an analogous process, using a gamma hazard function to reduce the probability of COs at DSBs located in nearby bins. CO selection continued until 64% of DSBs had been selected as COs. After CO selection all remaining DSBs were considered NCOs. To simulate failure to detect some events, 20% of all events were randomly deleted, and then 30% of the remaining NCOs were randomly deleted. Interference between all simulated events (before deletion of “undetectable” products) is reported as “DSBs” in Fig 6B. For Fig 6B, all four chromatids were treated as a single entity; i.e., DSB interference was applied equally to all four chromatids. Simulations of same-chromatid-only or intersister-only DSB interference are in S8B Fig. Scripts used to simulate tetrads and calculate interference have been deposited in Dryad Digital Repository (doi:10.5061/dryad.bj042).

### Modeling DSB distribution

The DSB landscape was incorporated into randomized tetrads by using DSB frequencies measured by sequencing of Spo11-oligos [69]. The genome was divided into non-overlapping bins of 2 kb, and the DSB signals for all nucleotide positions in each bin were added together and used to set the probability of events occurring in that bin. For analysis of complex event frequency in S8C and S8D Fig, bins within 10 kb of a telomere were not used because they contain lower-than-expected numbers of complex events; this is because the number of possible events for merging (within 5 kb) is limited on one side by the chromosome end.

## Supporting Information

S1 Fig Recombination in *tel1Δ*. Analysis was performed as in Fig 2, but without merging close events. A) The average number of COs, NCOs, and all events (COs + NCOs) per tetrad is shown. COs include event types E2, E3, E5, E6, and E7 as defined in Fig 3. NCOs include E1 and E4. B) The average ratio of COs to NCOs is shown for wt and *tel1Δ*. C) Histogram of distances between pairs of adjacent COs. D) Interference (1 –CoC) for COs in wild-type and *tel1Δ* tetrads. For each inter-interval distance, the CoC was calculated individually for all possible interval pairs genome-wide, and the average is plotted. For all plots, analysis of COs used data from 52 wild-type and 14 *tel1Δ* tetrads; analysis of NCOs and all events used data from 52 wild-type and eight *tel1Δ* tetrads. Error bars: standard error (SE).
(PDF)

S2 Fig Recombination products in the six strains shown in Fig 3. A) All event types contributing to the analysis in Fig 3C are shown in detail here. B) Analysis was performed as in A, but without merging close events.
(PDF)

S3 Fig Phenotypes of *tel1Δ* and *sgs1Δ*. A) Analysis was performed as in Fig 3B, but without merging close events. The average number of COs, NCOs, and all events (COs + NCOs) per tetrad is shown. COs include event types E2, E3, E5, E6, and E7 as defined in Fig 3. NCOs include E1 and E4. B) As in Fig 3C, but without merging close events. The average length of GC tracts at simple COs (E2) is shown. C) As in Fig 3D, but without merging close events. Histogram of the lengths of simple NCOs (E1). D) The average number of spores per ascus is shown for the same sporulations summarized in Fig 3E. “0 spores” indicates unsporulated cells. Three- and four-spore asci are reported as a single category because they cannot be reliably distinguished. Sporulation was measured in three independent cultures of each genotype, with the exception of *sgs1Δ* for which only two cultures were used. At least 300 cells per culture were counted. Error bars in all plots: SE. For plots A-D except analysis of COs in part A, data were derived from 52 wildtype, eight *tel1Δ*, nine *sgs1Δ*, seven *zip3Δ*, six *zip3Δ tel1Δ*, and six *zip3Δ sgs1Δ* tetrads. Analysis of CO frequency in part A used an additional set of six *tel1Δ*, four *sgs1Δ*, and 23 *zip3Δ* tetrads genotyped at lower resolution.
(PDF)

S4 Fig Zip3 focus data. A) Distances between pairs of adjacent Zip3 foci on chromosome IV. Data include 454 wild-type and 399 *tel1Δ* focus pairs. B) Areas of individual foci were determined after automated focus finding in ImageJ. Foci on all chromosomes are included. Bars: mean and standard deviation. P values: Student’s t test.
(PDF)

S5 Fig Zip3 focus and SC length measurements. A, B and C) Data pooled in Fig 4B, 4C and 4F, plotted here as individual experiments. Experiments 1, 2 and 5 used strains yCA1442 and yCA1443 (wt and *tel1Δ*, respectively) while Experiments 3 and 4 used strains yCA1444 and yCA1445 (wt and *tel1Δ*, respectively). The two pairs of strains are independent isolates of the same genotypes. A: Number of Zip3 foci on chromosome IV. B: Number of Zip3 foci per cell determined by automated focus finding in ImageJ, using the same images scored in A. C: Length of chromosome IV SC, visualized by Zip1 staining, also from the same set of images scored in A. Bars: mean and standard deviation. P values: Student’s t test.
(PDF)

S6 Fig Zip3 dependence of COs in *tel1Δ*. A) Analysis was performed as in Fig 5A, but without merging close events. The average number of Zip3-GFP foci on chromosome IV detected on spreads (as in Fig 4) divided by the average number of COs on chromosome IV in genotyped tetrads (as in S1A Fig). B) The average number of Zip2 foci on chromosome XV detected on spreads [9] divided by the average number of COs on chromosome XV in genotyped tetrads (this study and [50].) C) Analysis was performed as in Fig 5D, but without merging close events. The average number of COs genome wide is expressed as a percent of all interhomolog events genome wide. Per-tetrad averages are shown. D) The density of COs on each chromosome was calculated using merged events. Error bars: SE.
(PDF)

S7 Fig Loss of detection of some recombination events does not significantly alter CoC. Failure to detect some events was simulated using a data set consisting of all recombination products from 52 wild-type tetrads. At each sampling level, events were randomly removed from each tetrad until the indicated percent of events remained (for example, “80%” indicates that 20% of events were removed from each tetrad). Interference (1-CoC) was calculated based on the remaining events. This procedure was repeated 200 times at each sampling level and the averages are plotted. This analysis demonstrates that failure to detect some events does not significantly alter the estimate of interference as long as the detectable events reflect the underlying distribution of all events. B) Interference for an inter-interval distance of 25 kb is shown for the same data set (i.e., the first point from each curve in S7A Fig). Error bars: SE.
(PDF)

S8 Fig Distribution of events in *tel1Δ*, *sgs1Δ*, and *ZMM* mutants. A) Analysis was performed as in Fig 6A, but without merging close events. The coefficient of coincidence for a bin size and inter-interval distance of 25 kb is shown for COs only, NCOs only, or all events. B) Simulations were performed as in Fig 6B, in which an interfering population of DSBs was first created, and then COs were selected from the DSBs. COs were selected with additional interference. Remaining DSBs were considered NCOs. Failure to detect some events was simulated by removing 20% of all events and 30% of the remaining NCOs. Interference was then calculated as 1-CoC for a bin size and inter-interval distance of 25 kb. “All four chromatids”: simulated DSB interference was applied equally across all four chromatids. This is the same data set plotted in Fig 6B. “Each pair of sisters”: DSB interference only affected each chromatid and its sister. The strength of DSB and CO interference were selected to recapitulate the wild type levels of interference between COs and all detectable products. “Each chromatid”: simulated DSB interference only applied to DSBs on the same chromatid. In this simulation, it was not possible to recapitulate the wild type level of interference among all products even at extremely high levels of same-chromatid DSB interference. White bars: simulated strength of DSB interference when calculated between all four chromatids. Black bars: simulated strength of DSB interference when calculated along a single chromatid, a single pair of sisters, or all four chromatids, depending on which scenario was simulated .C and D) After randomization incorporating DSB frequencies (Fig 6C and 6D), the genome was divided into 2-kb bins and sorted into ten percentile ranges based on DSB frequency. For each percentile range, the percentage of products classified as complex or four-chromatid is plotted against the median DSB frequency of bins in that range. Error bars: SE.
(PDF)

S1 Table Yeast strains. (PDF)

S2 Table Tetrads genotyped. (PDF)

S1 Text Supporting materials and methods and supporting references. (PDF)

## References

[1] Berchowitz LE, Copenhaver GP (2010) Genetic interference: don't stand so close to me. Curr Genomics 11: 91–102. 10.2174/138920210790886835 20885817 PMC2874225

[2] Sturtevant AH (1913) A Third Group of Linked Genes in *Drosophila Ampelophila* . Science 37: 990–992. 17833164 10.1126/science.37.965.990

[3] Keeney S (2008) Spo11 and the Formation of DNA Double-Strand Breaks in Meiosis. Genome Dyn Stab 2: 81–123. 21927624 10.1007/7050_2007_026 PMC3172816

[4] Allers T, Lichten M (2001) Differential timing and control of noncrossover and crossover recombination during meiosis. Cell 106: 47–57. 11461701 10.1016/s0092-8674(01)00416-0

[5] Borner GV, Kleckner N, Hunter N (2004) Crossover/noncrossover differentiation, synaptonemal complex formation, and regulatory surveillance at the leptotene/zygotene transition of meiosis. Cell 117: 29–45. 15066280 10.1016/s0092-8674(04)00292-2

[6] Hunter N, Kleckner N (2001) The single-end invasion: an asymmetric intermediate at the double-strand break to double-Holliday junction transition of meiotic recombination. Cell 106: 59–70. 11461702 10.1016/s0092-8674(01)00430-5

[7] Agarwal S, Roeder GS (2000) Zip3 provides a link between recombination enzymes and synaptonemal complex proteins. Cell 102: 245–255. 10943844 10.1016/s0092-8674(00)00029-5

[8] Chua PR, Roeder GS (1998) Zip2, a meiosis-specific protein required for the initiation of chromosome synapsis. Cell 93: 349–359. 9590170 10.1016/s0092-8674(00)81164-2

[9] Fung JC, Rockmill B, Odell M, Roeder GS (2004) Imposition of crossover interference through the nonrandom distribution of synapsis initiation complexes. Cell 116: 795–802. 15035982 10.1016/s0092-8674(04)00249-1

[10] Henderson KA, Keeney S (2004) Tying synaptonemal complex initiation to the formation and programmed repair of DNA double-strand breaks. Proc Natl Acad Sci U S A 101: 4519–4524. 15070750 10.1073/pnas.0400843101 PMC384779

[11] Serrentino ME, Chaplais E, Sommermeyer V, Borde V (2013) Differential association of the conserved SUMO ligase Zip3 with meiotic double-strand break sites reveals regional variations in the outcome of meiotic recombination. PLoS Genet 9: e1003416 10.1371/journal.pgen.1003416 23593021 PMC3616913

[12] Lynn A, Soucek R, Borner GV (2007) ZMM proteins during meiosis: crossover artists at work. Chromosome Res 15: 591–605. 17674148 10.1007/s10577-007-1150-1

[13] Wang S, Zickler D, Kleckner N, Zhang L (2015) Meiotic crossover patterns: obligatory crossover, interference and homeostasis in a single process. Cell Cycle 14: 305–314. 10.4161/15384101.2014.991185 25590558 PMC4353236

[14] Zhang L, Espagne E, de Muyt A, Zickler D, Kleckner NE (2014) Interference-mediated synaptonemal complex formation with embedded crossover designation. Proc Natl Acad Sci U S A 111: E5059–5068. 10.1073/pnas.1416411111 25380597 PMC4250137

[15] Zhang L, Liang Z, Hutchinson J, Kleckner N (2014) Crossover patterning by the beam-film model: analysis and implications. PLoS Genet 10: e1004042 10.1371/journal.pgen.1004042 24497834 PMC3907302

[16] Zhang L, Wang S, Yin S, Hong S, Kim KP, Kleckner N (2014) Topoisomerase II mediates meiotic crossover interference. Nature 511: 551–556. 10.1038/nature13442 25043020 PMC4128387

[17] Joshi N, Barot A, Jamison C, Borner GV (2009) Pch2 links chromosome axis remodeling at future crossover sites and crossover distribution during yeast meiosis. PLoS Genet 5: e1000557 10.1371/journal.pgen.1000557 19629172 PMC2708914

[18] Lao JP, Cloud V, Huang CC, Grubb J, Thacker D, Lee CY, et al (2013) Meiotic crossover control by concerted action of Rad51-Dmc1 in homolog template bias and robust homeostatic regulation. PLoS Genet 9: e1003978 10.1371/journal.pgen.1003978 24367271 PMC3868528

[19] Lisby M, Rothstein R (2009) Choreography of recombination proteins during the DNA damage response. DNA Repair (Amst) 8: 1068–1076. 19473884 10.1016/j.dnarep.2009.04.007 PMC2729071

[20] Lange J, Pan J, Cole F, Thelen MP, Jasin M, Keeney S (2011) ATM controls meiotic double-strand-break formation. Nature 479: 237–240. 10.1038/nature10508 22002603 PMC3213282

[21] Joyce EF, Pedersen M, Tiong S, White-Brown SK, Paul A, Campbell SD, et al (2011) *Drosophila* ATM and ATR have distinct activities in the regulation of meiotic DNA damage and repair. J Cell Biol 195: 359–367. 10.1083/jcb.201104121 22024169 PMC3206348

[22] Carballo JA, Panizza S, Serrentino ME, Johnson AL, Geymonat M, Borde V, et al (2013) Budding yeast ATM/ATR control meiotic double-strand break (DSB) levels by down-regulating Rec114, an essential component of the DSB-machinery. PLoS Genet 9: e1003545 10.1371/journal.pgen.1003545 23825959 PMC3694840

[23] Garcia V, Gray S, Allison RM, Cooper TJ, Neale MJ (2015) Tel1-mediated interference suppresses clustered meiotic double-strand-break formation. Nature 520: 114–118. 10.1038/nature13993 25539084 PMC7116500

[24] Zhang L, Kim KP, Kleckner NE, Storlazzi A (2011) Meiotic double-strand breaks occur once per pair of (sister) chromatids and, via Mec1/ATR and Tel1/ATM, once per quartet of chromatids. Proc Natl Acad Sci U S A 108: 20036–20041. 10.1073/pnas.1117937108 22123968 PMC3250133

[25] Argunhan B, Farmer S, Leung WK, Terentyev Y, Humphryes N, Tsubouchi T, et al (2013) Direct and indirect control of the initiation of meiotic recombination by DNA damage checkpoint mechanisms in budding yeast. PLoS One 8: e65875 10.1371/journal.pone.0065875 23762445 PMC3677890

[26] Blitzblau HG, Hochwagen A (2013) ATR/Mec1 prevents lethal meiotic recombination initiation on partially replicated chromosomes in budding yeast. Elife 2: e00844 10.7554/eLife.00844 24137535 PMC3787542

[27] Barchi M, Mahadevaiah S, Di Giacomo M, Baudat F, de Rooij DG, Burgoyne PS, et al (2005) Surveillance of different recombination defects in mouse spermatocytes yields distinct responses despite elimination at an identical developmental stage. Mol Cell Biol 25: 7203–7215. 16055729 10.1128/MCB.25.16.7203-7215.2005 PMC1190256

[28] Barlow C, Liyanage M, Moens PB, Tarsounas M, Nagashima K, Brown K, et al (1998) Atm deficiency results in severe meiotic disruption as early as leptonema of prophase I. Development 125: 4007–4017. 9735362 10.1242/dev.125.20.4007

[29] Di Giacomo M, Barchi M, Baudat F, Edelmann W, Keeney S, Jasin M (2005) Distinct DNA-damage-dependent and -independent responses drive the loss of oocytes in recombination-defective mouse mutants. Proc Natl Acad Sci U S A 102: 737–742. 15640358 10.1073/pnas.0406212102 PMC545532

[30] Barchi M, Roig I, Di Giacomo M, de Rooij DG, Keeney S, Jasin M (2008) ATM promotes the obligate XY crossover and both crossover control and chromosome axis integrity on autosomes. PLoS Genet 4: e1000076 10.1371/journal.pgen.1000076 18497861 PMC2374915

[31] Bellani MA, Romanienko PJ, Cairatti DA, Camerini-Otero RD (2005) SPO11 is required for sex-body formation, and *Spo11* heterozygosity rescues the prophase arrest of *Atm−/−* spermatocytes. J Cell Sci 118: 3233–3245. 15998665 10.1242/jcs.02466

[32] Joshi N, Brown MS, Bishop DK, Borner GV (2015) Gradual implementation of the meiotic recombination program via checkpoint pathways controlled by global DSB levels. Mol Cell 57: 797–811. 10.1016/j.molcel.2014.12.027 25661491 PMC4392720

[33] Cooper TJ, Wardell K, Garcia V, Neale MJ (2014) Homeostatic regulation of meiotic DSB formation by ATM/ATR. Exp Cell Res 329: 124–131. 10.1016/j.yexcr.2014.07.016 25116420

[34] Fan QQ, Xu F, White MA, Petes TD (1997) Competition between adjacent meiotic recombination hotspots in the yeast *Saccharomyces cerevisiae* . Genetics 145: 661–670. 9055076 10.1093/genetics/145.3.661 PMC1207851

[35] Robine N, Uematsu N, Amiot F, Gidrol X, Barillot E, Nicolas A, et al (2007) Genome-wide redistribution of meiotic double-strand breaks in *Saccharomyces cerevisiae* . Mol Cell Biol 27: 1868–1880. 17189430 10.1128/MCB.02063-06 PMC1820458

[36] Wu TC, Lichten M (1995) Factors that affect the location and frequency of meiosis-induced double-strand breaks in *Saccharomyces cerevisiae* . Genetics 140: 55–66. 7635308 10.1093/genetics/140.1.55 PMC1206571

[37] Xu L, Kleckner N (1995) Sequence non-specific double-strand breaks and interhomolog interactions prior to double-strand break formation at a meiotic recombination hot spot in yeast. EMBO J 14: 5115–5128. 7588640 10.1002/j.1460-2075.1995.tb00194.x PMC394615

[38] Carballo JA, Johnson AL, Sedgwick SG, Cha RS (2008) Phosphorylation of the axial element protein Hop1 by Mec1/Tel1 ensures meiotic interhomolog recombination. Cell 132: 758–770. 10.1016/j.cell.2008.01.035 18329363

[39] Cartagena-Lirola H, Guerini I, Viscardi V, Lucchini G, Longhese MP (2006) Budding Yeast Sae2 is an In Vivo Target of the Mec1 and Tel1 Checkpoint Kinases During Meiosis. Cell Cycle 5: 1549–1559. 16861895 10.4161/cc.5.14.2916

[40] Cheng YH, Chuang CN, Shen HJ, Lin FM, Wang TF (2013) Three distinct modes of Mec1/ATR and Tel1/ATM activation illustrate differential checkpoint targeting during budding yeast early meiosis. Mol Cell Biol 33: 3365–3376. 10.1128/MCB.00438-13 23775120 PMC3753904

[41] Ho HC, Burgess SM (2011) Pch2 acts through Xrs2 and Tel1/ATM to modulate interhomolog bias and checkpoint function during meiosis. PLoS Genet 7: e1002351 10.1371/journal.pgen.1002351 22072981 PMC3207854

[42] Keeney S, Lange J, Mohibullah N (2014) Self-organization of meiotic recombination initiation: general principles and molecular pathways. Annu Rev Genet 48: 187–214. 10.1146/annurev-genet-120213-092304 25421598 PMC4291115

[43] Henzel JV, Nabeshima K, Schvarzstein M, Turner BE, Villeneuve AM, Hillers KJ (2011) An asymmetric chromosome pair undergoes synaptic adjustment and crossover redistribution during *Caenorhabditis elegans* meiosis: implications for sex chromosome evolution. Genetics 187: 685–699. 10.1534/genetics.110.124958 21212235 PMC3063665

[44] Nabeshima K, Villeneuve AM, Hillers KJ (2004) Chromosome-wide regulation of meiotic crossover formation in *Caenorhabditis elegans* requires properly assembled chromosome axes. Genetics 168: 1275–1292. 15579685 10.1534/genetics.104.030700 PMC1448768

[45] Hayashi M, Mlynarczyk-Evans S, Villeneuve AM (2010) The synaptonemal complex shapes the crossover landscape through cooperative assembly, crossover promotion and crossover inhibition during *Caenorhabditis elegans* meiosis. Genetics 186: 45–58. 10.1534/genetics.110.115501 20592266 PMC2940310

[46] Kauppi L, Barchi M, Lange J, Baudat F, Jasin M, Keeney S (2013) Numerical constraints and feedback control of double-strand breaks in mouse meiosis. Genes Dev 27: 873–886. 10.1101/gad.213652.113 23599345 PMC3650225

[47] Thacker D, Mohibullah N, Zhu X, Keeney S (2014) Homologue engagement controls meiotic DNA break number and distribution. Nature 510: 241–246. 10.1038/nature13120 24717437 PMC4057310

[48] Wojtasz L, Daniel K, Roig I, Bolcun-Filas E, Xu H, Boonsanay V, et al (2009) Mouse HORMAD1 and HORMAD2, two conserved meiotic chromosomal proteins, are depleted from synapsed chromosome axes with the help of TRIP13 AAA-ATPase. PLoS Genet 5: e1000702 10.1371/journal.pgen.1000702 19851446 PMC2758600

[49] Anderson CM, Chen SY, Dimon MT, Oke A, DeRisi JL, Fung JC (2011) ReCombine: a suite of programs for detection and analysis of meiotic recombination in whole-genome datasets. PLoS One 6: e25509 10.1371/journal.pone.0025509 22046241 PMC3201961

[50] Chen SY, Tsubouchi T, Rockmill B, Sandler JS, Richards DR, Vader G, et al (2008) Global analysis of the meiotic crossover landscape. Dev Cell 15: 401–415. 10.1016/j.devcel.2008.07.006 18691940 PMC2628562

[51] Mancera E, Bourgon R, Brozzi A, Huber W, Steinmetz LM (2008) High-resolution mapping of meiotic crossovers and non-crossovers in yeast. Nature 454: 479–485. 10.1038/nature07135 18615017 PMC2780006

[52] Qi J, Wijeratne AJ, Tomsho LP, Hu Y, Schuster SC, Ma H (2009) Characterization of meiotic crossovers and gene conversion by whole-genome sequencing in *Saccharomyces cerevisiae* . BMC Genomics 10: 475 10.1186/1471-2164-10-475 19832984 PMC2770529

[53] Oke A, Anderson CM, Yam P, Fung JC (2014) Controlling Meiotic Recombinational Repair—Specifying the Roles of ZMMs, Sgs1 and Mus81/Mms4 in Crossover Formation. PLoS Genet 10: e1004690 10.1371/journal.pgen.1004690 25329811 PMC4199502

[54] Jessop L, Rockmill B, Roeder GS, Lichten M (2006) Meiotic chromosome synapsis-promoting proteins antagonize the anti-crossover activity of Sgs1. PLoS Genet 2: e155 17002499 10.1371/journal.pgen.0020155 PMC1570379

[55] Oh SD, Lao JP, Hwang PY, Taylor AF, Smith GR, Hunter N (2007) BLM ortholog, Sgs1, prevents aberrant crossing-over by suppressing formation of multichromatid joint molecules. Cell 130: 259–272. 17662941 10.1016/j.cell.2007.05.035 PMC2034285

[56] Rockmill B, Fung JC, Branda SS, Roeder GS (2003) The Sgs1 helicase regulates chromosome synapsis and meiotic crossing over. Curr Biol 13: 1954–1962. 14614820 10.1016/j.cub.2003.10.059

[57] Libuda DE, Uzawa S, Meyer BJ, Villeneuve AM (2013) Meiotic chromosome structures constrain and respond to designation of crossover sites. Nature 502: 703–706. 10.1038/nature12577 24107990 PMC3920622

[58] Lynn A, Koehler KE, Judis L, Chan ER, Cherry JP, Schwartz S, et al (2002) Covarian of synaptonemal complex length and mammalian meiotic exchange rates. Science 296: 22222225. 10.1126/science.1071220 12052900

[59] Mets DG, Meyer BJ (2009) Condensins regulate meiotic DNA break distribution, thus crossover frequency, by controlling chromosome structure. Cell 139: 73–86. 10.1016/j.cell.2009.07.035 19781752 PMC2785808

[60] Yokoo R, Zawadzki KA, Nabeshima K, Drake M, Arur S, Villeneuve AM (2012) COSA-1 reveals robust homeostasis and separable licensing and reinforcement steps governing meiotic crossovers. Cell 149: 75–87. 10.1016/j.cell.2012.01.052 22464324 PMC3339199

[61] Youds JL, Mets DG, McIlwraith MJ, Martin JS, Ward JD, NJ ON, et al (2010) RTEL-1 enforces meiotic crossover interference and homeostasis. Science 327: 1254–1258. 10.1126/science.1183112 20203049 PMC4770885

[62] Terasawa M, Ogawa T, Tsukamoto Y, Ogawa H (2008) Sae2p phosphorylation is crucial for cooperation with Mre11p for resection of DNA double-strand break ends during meiotic recombination in *Saccharomyces cerevisiae* . Genes Genet Syst 83: 209–217. 18670132 10.1266/ggs.83.209

[63] de los Santos T, Hunter N, Lee C, Larkin B, Loidl J, Hollingsworth NM (2003) The Mus81/Mms4 endonuclease acts independently of double-Holliday junction resolution to promote a distinct subset of crossovers during meiosis in budding yeast. Genetics 164: 81–94. 12750322 10.1093/genetics/164.1.81 PMC1462551

[64] Stahl FW, Foss HM, Young LS, Borts RH, Abdullah MF, Copenhaver GP (2004) Does crossover interference count in *Saccharomyces cerevisiae*? Genetics 168: 35–48. 15454525 10.1534/genetics.104.027789 PMC1448104

[65] Zalevsky J, MacQueen AJ, Duffy JB, Kemphues KJ, Villeneuve AM (1999) Crossing over during *Caenorhabditis elegans* meiosis requires a conserved MutS-based pathway that is partially dispensable in budding yeast. Genetics 153: 1271–1283. 10545458 10.1093/genetics/153.3.1271 PMC1460811

[66] Schwacha A, Kleckner N (1997) Interhomolog bias during meiotic recombination: meiotic functions promote a highly differentiated interhomolog-only pathway. Cell 90: 1123–1135. 9323140 10.1016/s0092-8674(00)80378-5

[67] Gray S, Allison RM, Garcia V, Goldman AS, Neale MJ (2013) Positive regulation of meiotic DNA double-strand break formation by activation of the DNA damage checkpoint kinase Mec1(ATR). Open Biol 3: 130019 10.1098/rsob.130019 23902647 PMC3728922

[68] Rockmill B, Lefrancois P, Voelkel-Meiman K, Oke A, Roeder GS, Fung JC (2013) High throughput sequencing reveals alterations in the recombination signatures with diminishing Spo11 activity. PLoS Genet 9: e1003932 10.1371/journal.pgen.1003932 24204324 PMC3814317

[69] Pan J, Sasaki M, Kniewel R, Murakami H, Blitzblau HG, Tischfield SE, et al (2011) A hierarchical combination of factors shapes the genome-wide topography of yeast meiotic recombination initiation. Cell 144: 719–731. 10.1016/j.cell.2011.02.009 21376234 PMC3063416

[70] Craven RJ, Greenwell PW, Dominska M, Petes TD (2002) Regulation of genome stability by *TEL1* and *MEC1*, yeast homologs of the mammalian *ATM* and *ATR* genes. Genetics 161: 493–507. 12072449 10.1093/genetics/161.2.493 PMC1462148

[71] Greenwell PW, Kronmal SL, Porter SE, Gassenhuber J, Obermaier B, Petes TD (1995) *TEL1*, a gene involved in controlling telomere length in S. cerevisiae, is homologous to the human ataxia telangiectasia gene. Cell 82: 823–829. 7671310 10.1016/0092-8674(95)90479-4

[72] Suetomi K, Mochizuki M, Suzuki S, Yamamoto H, Yamamoto K (2010) Effects of *Saccharomyces cerevisiae mec1*, *tel1*, and *mre11* mutations on spontaneous and methylmethane sulfonate-induced genome instability. Genes Genet Syst 85: 1–8. 20410660 10.1266/ggs.85.1

[73] Anderson LK, Reeves A, Webb LM, Ashley T (1999) Distribution of crossing over on mouse synaptonemal complexes using immunofluorescent localization of MLH1 protein. Genetics 151: 1569–1579. 10101178 10.1093/genetics/151.4.1569 PMC1460565

[74] Falque M, Mercier R, Mezard C, de Vienne D, Martin OC (2007) Patterns of recombination and MLH1 foci density along mouse chromosomes: modeling effects of interference and obligate chiasma. Genetics 176: 1453–1467. 17483430 10.1534/genetics.106.070235 PMC1931555

[75] Holloway JK, Booth J, Edelmann W, McGowan CH, Cohen PE (2008) MUS81 generates a subset of MLH1-MLH3-independent crossovers in mammalian meiosis. PLoS Genet 4: e1000186 10.1371/journal.pgen.1000186 18787696 PMC2525838

[76] Svetlanov A, Baudat F, Cohen PE, de Massy B (2008) Distinct functions of MLH3 at recombination hot spots in the mouse. Genetics 178: 1937–1945. 10.1534/genetics.107.084798 18430927 PMC2323788

[77] Buhler C, Borde V, Lichten M (2007) Mapping meiotic single-strand DNA reveals a new landscape of DNA double-strand breaks in *Saccharomyces cerevisiae* . PLoS Biol 5: e324 10.1371/journal.pbio.0060104 18076285 PMC2121111

[78] Rockmill B (2009) Chromosome spreading and immunofluorescence methods in *Saccharomyces cerevisiae* . Methods Mol Biol 558: 3–13. 10.1007/978-1-60761-103-5_1 19685315

