# Correction: Reduced crossover interference and increased ZMM-independent recombination in the absence of Tel1/ATM

**DOI:** 10.1371/journal.pgen.1011779

**Published:** 2025-07-07

**Authors:** Carol M. Anderson, Ashwini Oke, Phoebe Yam, Tangna Zhuge, Jennifer C. Fung

The Data Availability statement for this article includes the incorrect Dryad information. The analysis files can be accessed from Dryad via the hyperlink: https://doi.org/10.5061/dryad.bj042.
