# Supplementary figures and images for: Reduced Crossover Interference and Increased ZMM-Independent Recombination in the Absence of Tel1/ATM
Source: PLoS Genet. 2015 Aug 25;11(8):e1005478. doi: 10.1371/journal.pgen.1005478 (PMC4549261; doi:10.1371/journal.pgen.1005478)

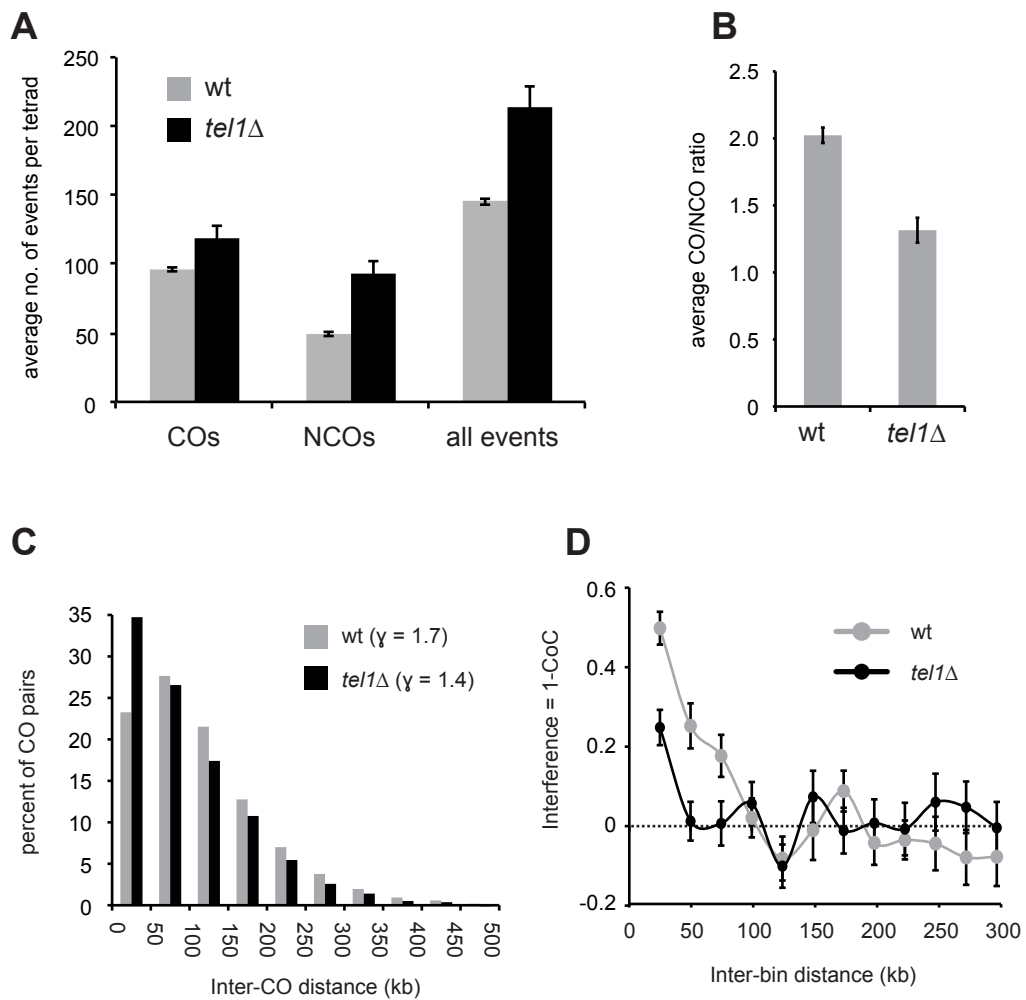

Supplement: S1 Fig — Analysis was performed as in Fig 2, but without merging close events. A) The average number of COs, NCOs, and all events (COs + NCOs) per tetrad is shown. COs include event types E2, E3, E5, E6, and E7 as defined in Fig 3. NCOs include E1 and E4. B) The average ratio of COs to NCOs is shown for wt and tel1Δ. C) Histogram of distances between pairs of adjacent COs. D) Interference (1 –CoC) for COs in wild-type and tel1Δ tetrads. For each inter-interval distance, the CoC was calculated individually for all possible interval pairs genome-wide, and the average is plotted. For all plots, analysis of COs used data from 52 wild-type and 14 tel1Δ tetrads; analysis of NCOs and all events used data from 52 wild-type and eight tel1Δ tetrads. Error bars: standard error (SE). (PDF) [file pgen.1005478.s001.pdf]

## A Events within 5 kb merged

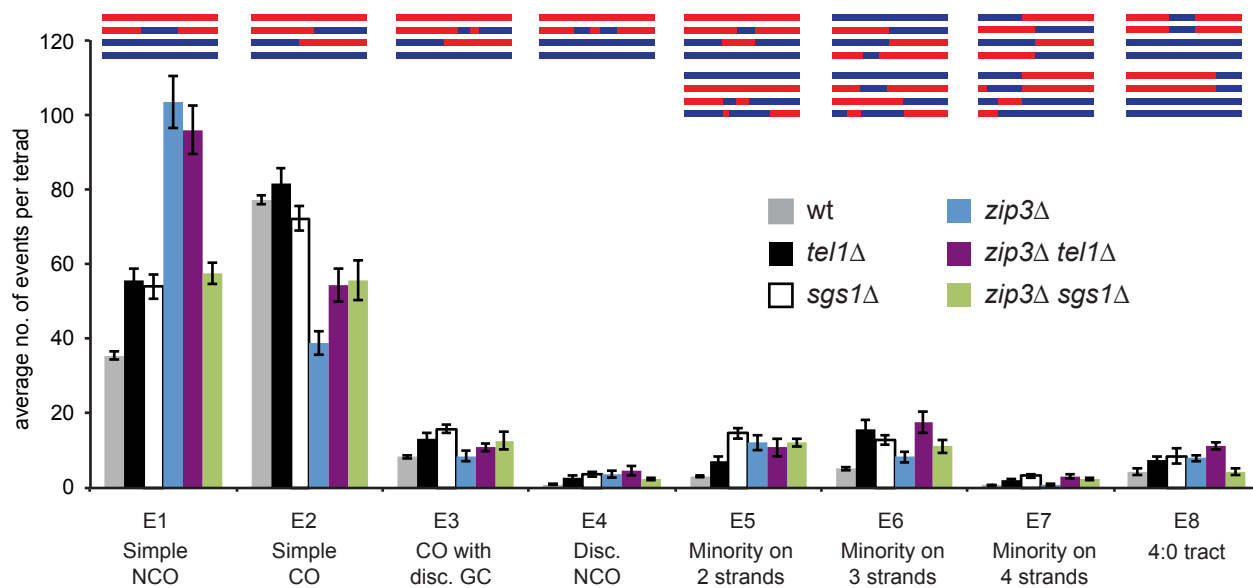

## B Unmerged events

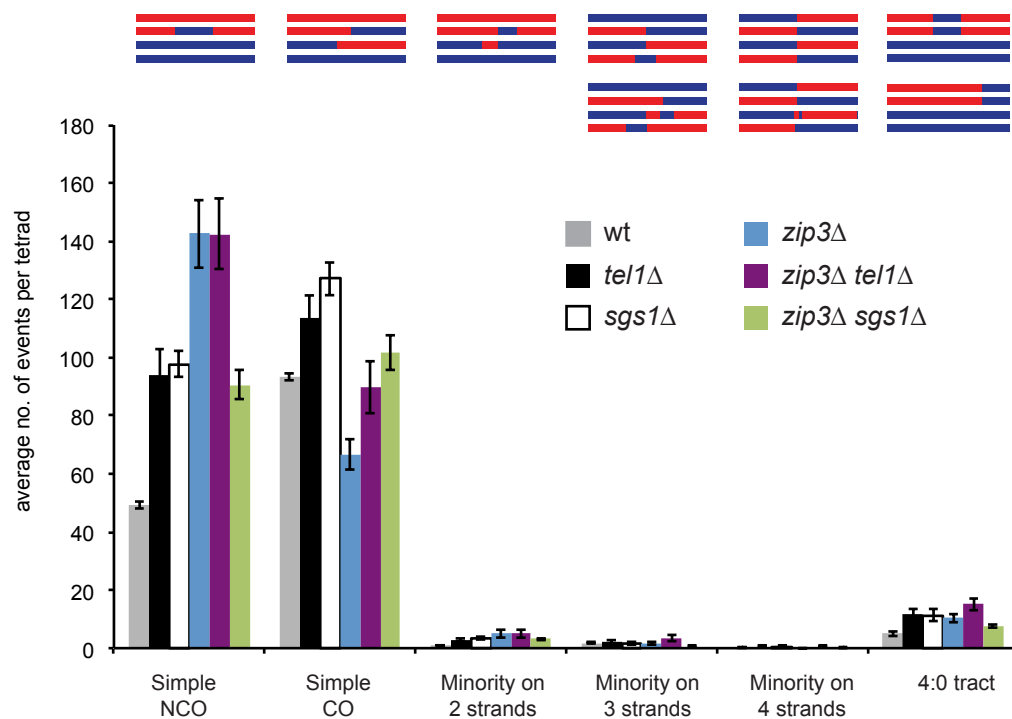

Supplement: S2 Fig — A) All event types contributing to the analysis in Fig 3C are shown in detail here. B) Analysis was performed as in A, but without merging close events. (PDF) [file pgen.1005478.s002.pdf]

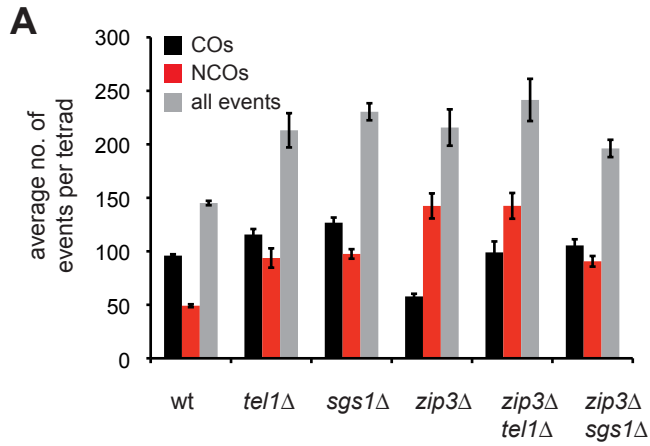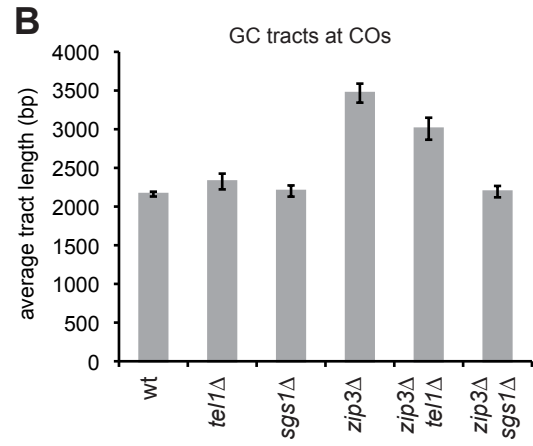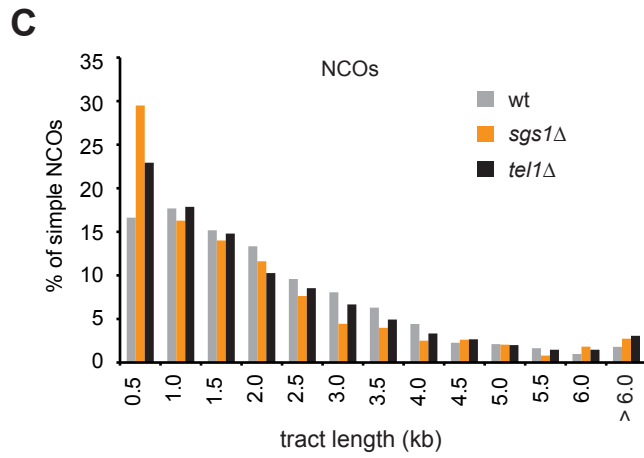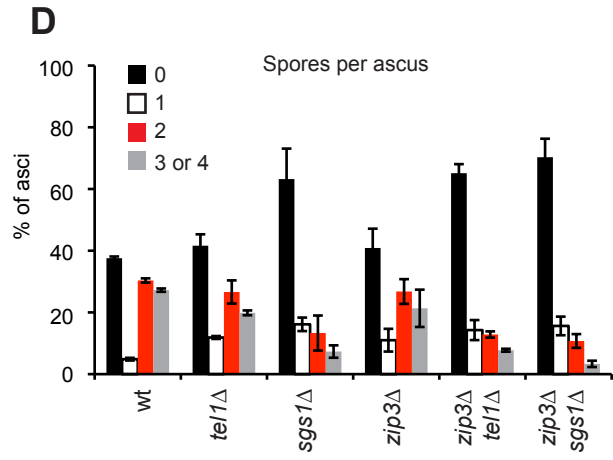

Supplement: S3 Fig — A) Analysis was performed as in Fig 3B, but without merging close events. The average number of COs, NCOs, and all events (COs + NCOs) per tetrad is shown. COs include event types E2, E3, E5, E6, and E7 as defined in Fig 3. NCOs include E1 and E4. B) As in Fig 3C, but without merging close events. The average length of GC tracts at simple COs (E2) is shown. C) As in Fig 3D, but without merging close events. Histogram of the lengths of simple NCOs (E1). D) The average number of spores per ascus is shown for the same sporulations summarized in Fig 3E. “0 spores” indicates unsporulated cells. Three- and four-spore asci are reported as a single category because they cannot be reliably distinguished. Sporulation was measured in three independent cultures of each genotype, with the exception of sgs1Δ for which only two cultures were used. At least 300 cells per culture were counted. Error bars in all plots: SE. For plots A-D except analysis of COs in part A, data were derived from 52 wildtype, eight tel1Δ, nine sgs1Δ, seven zip3Δ, six zip3Δ tel1Δ, and six zip3Δ sgs1Δ tetrads. Analysis of CO frequency in part A used an additional set of six tel1Δ, four sgs1Δ, and 23 zip3Δ tetrads genotyped at lower resolution. (PDF) [file pgen.1005478.s003.pdf]

**A**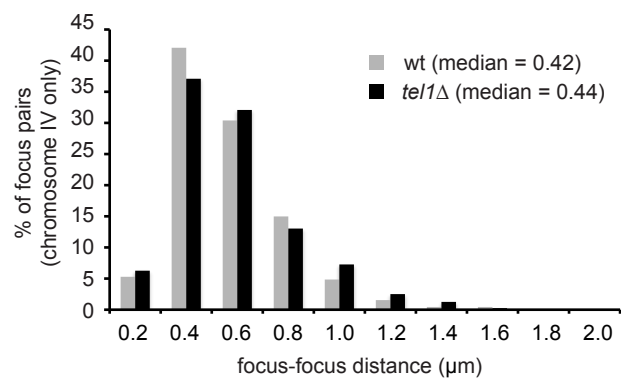**B**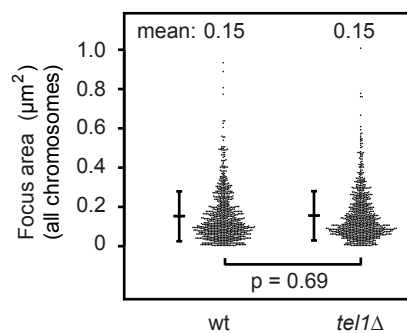

Supplement: S4 Fig — A) Distances between pairs of adjacent Zip3 foci on chromosome IV. Data include 454 wild-type and 399 tel1Δ focus pairs. B) Areas of individual foci were determined after automated focus finding in ImageJ. Foci on all chromosomes are included. Bars: mean and standard deviation. P values: Student’s t test. (PDF) [file pgen.1005478.s004.pdf]

**A**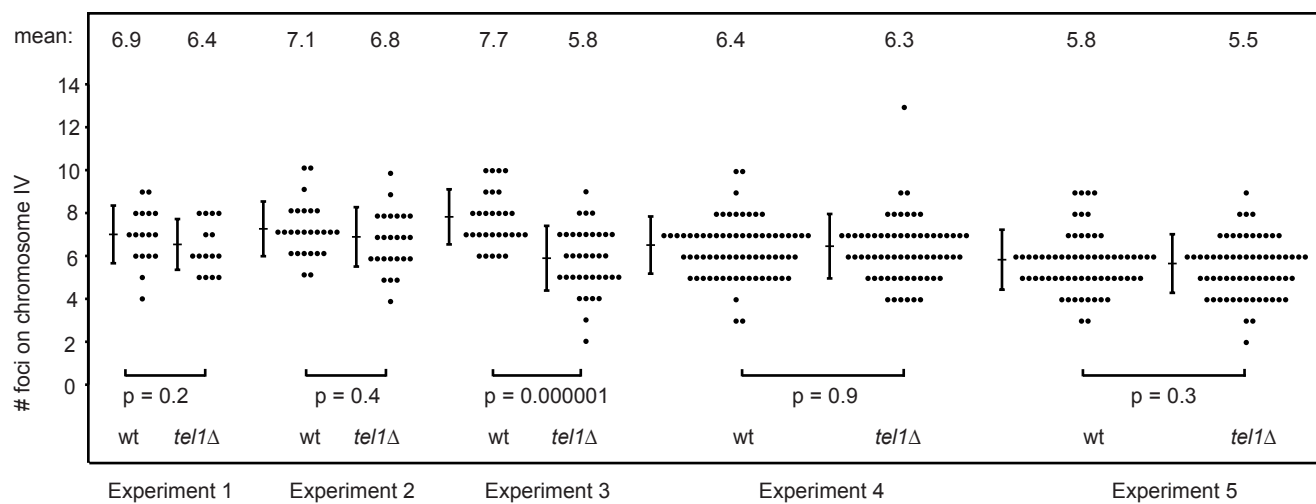**B**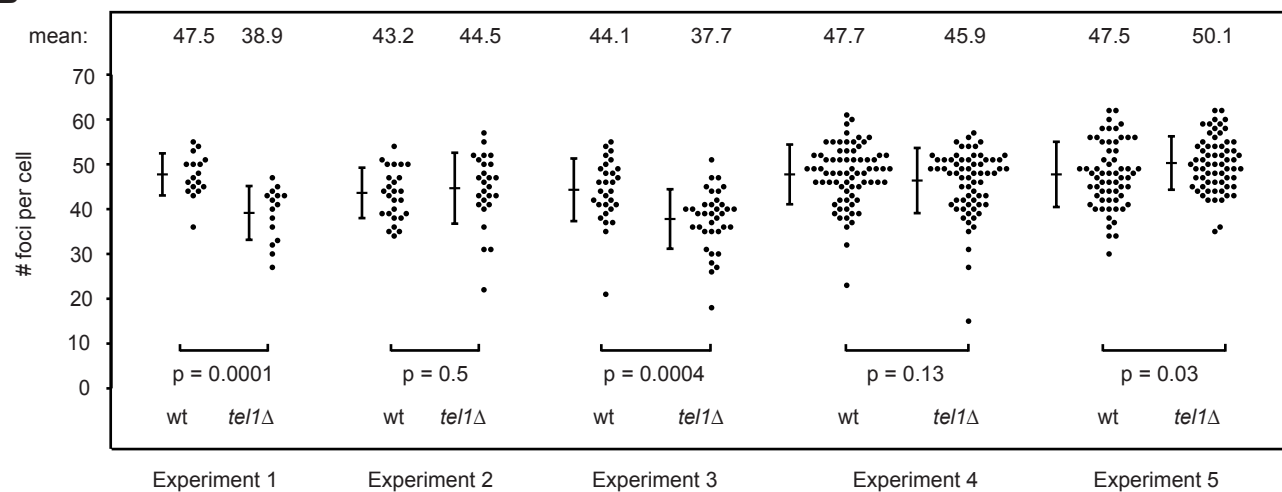**C**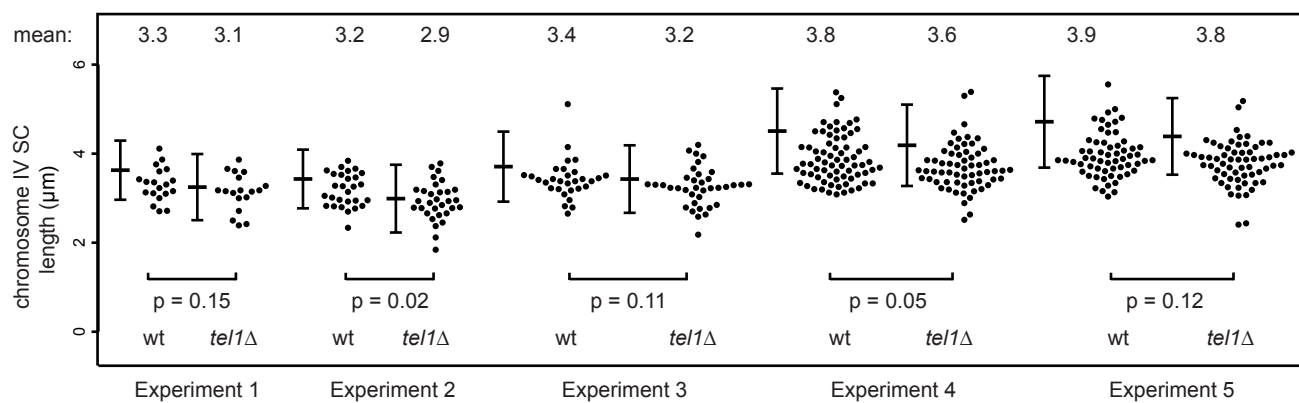

Supplement: S5 Fig — A, B and C) Data pooled in Fig 4B, 4C and 4F, plotted here as individual experiments. Experiments 1, 2 and 5 used strains yCA1442 and yCA1443 (wt and tel1Δ, respectively) while Experiments 3 and 4 used strains yCA1444 and yCA1445 (wt and tel1Δ, respectively). The two pairs of strains are independent isolates of the same genotypes. A: Number of Zip3 foci on chromosome IV. B: Number of Zip3 foci per cell determined by automated focus finding in ImageJ, using the same images scored in A. C: Length of chromosome IV SC, visualized by Zip1 staining, also from the same set of images scored in A. Bars: mean and standard deviation. P values: Student’s t test. (PDF) [file pgen.1005478.s005.pdf]

**A**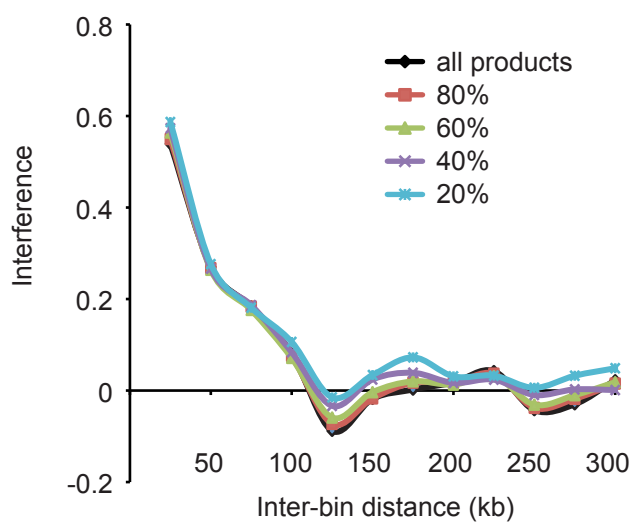**B**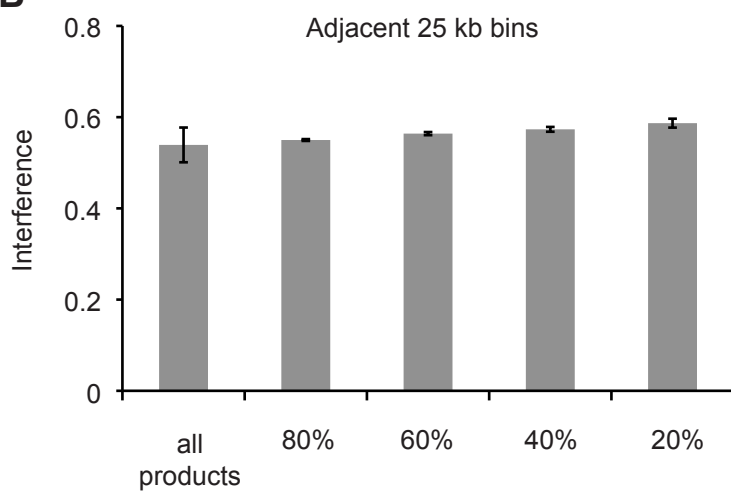

Supplement: S7 Fig — Failure to detect some events was simulated using a data set consisting of all recombination products from 52 wild-type tetrads. At each sampling level, events were randomly removed from each tetrad until the indicated percent of events remained (for example, “80%” indicates that 20% of events were removed from each tetrad). Interference (1-CoC) was calculated based on the remaining events. This procedure was repeated 200 times at each sampling level and the averages are plotted. This analysis demonstrates that failure to detect some events does not significantly alter the estimate of interference as long as the detectable events reflect the underlying distribution of all events. B) Interference for an inter-interval distance of 25 kb is shown for the same data set (i.e., the first point from each curve in S7A Fig). Error bars: SE. (PDF) [file pgen.1005478.s007.pdf]

**A**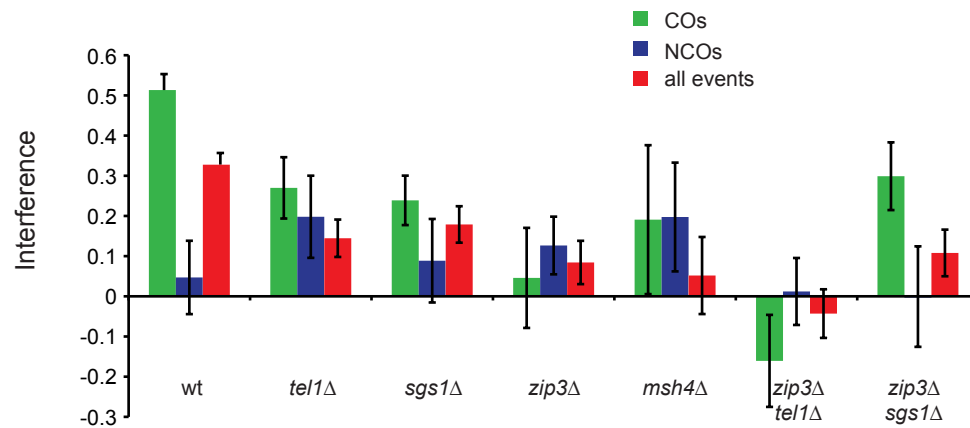**B**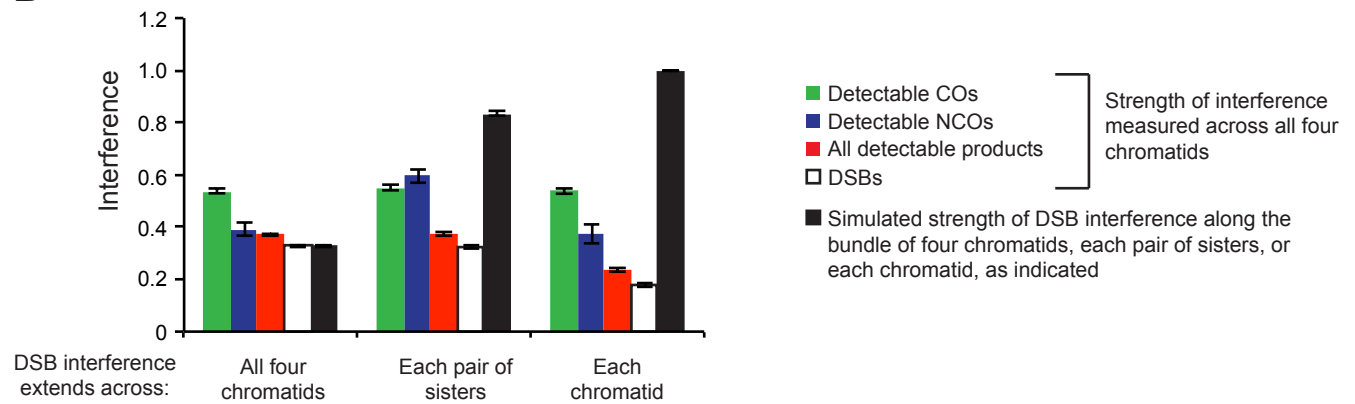**C**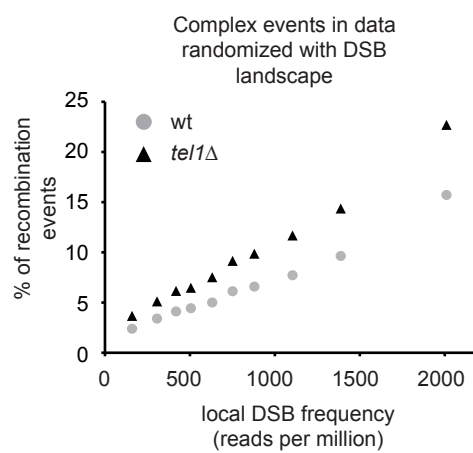**D**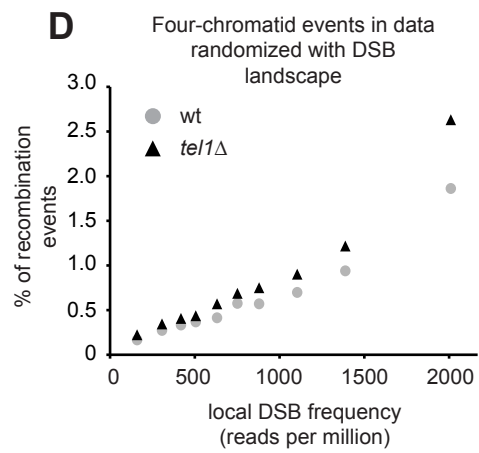

Supplement: S8 Fig — A) Analysis was performed as in Fig 6A, but without merging close events. The coefficient of coincidence for a bin size and inter-interval distance of 25 kb is shown for COs only, NCOs only, or all events. B) Simulations were performed as in Fig 6B, in which an interfering population of DSBs was first created, and then COs were selected from the DSBs. COs were selected with additional interference. Remaining DSBs were considered NCOs. Failure to detect some events was simulated by removing 20% of all events and 30% of the remaining NCOs. Interference was then calculated as 1-CoC for a bin size and inter-interval distance of 25 kb. “All four chromatids”: simulated DSB interference was applied equally across all four chromatids. This is the same data set plotted in Fig 6B. “Each pair of sisters”: DSB interference only affected each chromatid and its sister. The strength of DSB and CO interference were selected to recapitulate the wild type levels of interference between COs and all detectable products. “Each chromatid”: simulated DSB interference only applied to DSBs on the same chromatid. In this simulation, it was not possible to recapitulate the wild type level of interference among all products even at extremely high levels of same-chromatid DSB interference. White bars: simulated strength of DSB interference when calculated between all four chromatids. Black bars: simulated strength of DSB interference when calculated along a single chromatid, a single pair of sisters, or all four chromatids, depending on which scenario was simulated .C and D) After randomization incorporating DSB frequencies (Fig 6C and 6D), the genome was divided into 2-kb bins and sorted into ten percentile ranges based on DSB frequency. For each percentile range, the percentage of products classified as complex or four-chromatid is plotted against the median DSB frequency of bins in that range. Error bars: SE. (PDF) [file pgen.1005478.s008.pdf]
