# Supplementary material for: Reduced Crossover Interference and Increased ZMM-Independent Recombination in the Absence of Tel1/ATM
Source: PLoS Genet. 2015 Aug 25;11(8):e1005478. doi: 10.1371/journal.pgen.1005478 (PMC4549261; doi:10.1371/journal.pgen.1005478)
Supplement: S6 Fig — A) Analysis was performed as in Fig 5A, but without merging close events. The average number of Zip3-GFP foci on chromosome IV detected on spreads (as in Fig 4) divided by the average number of COs on chromosome IV in genotyped tetrads (as in S1A Fig). B) The average number of Zip2 foci on chromosome XV detected on spreads [9] divided by the average number of COs on chromosome XV in genotyped tetrads (this study and [50].) C) Analysis was performed as in Fig 5D, but without merging close events. The average number of COs genome wide is expressed as a percent of all interhomolog events genome wide. Per-tetrad averages are shown. D) The density of COs on each chromosome was calculated using merged events. Error bars: SE. (PDF) [file pgen.1005478.s006.pdf]

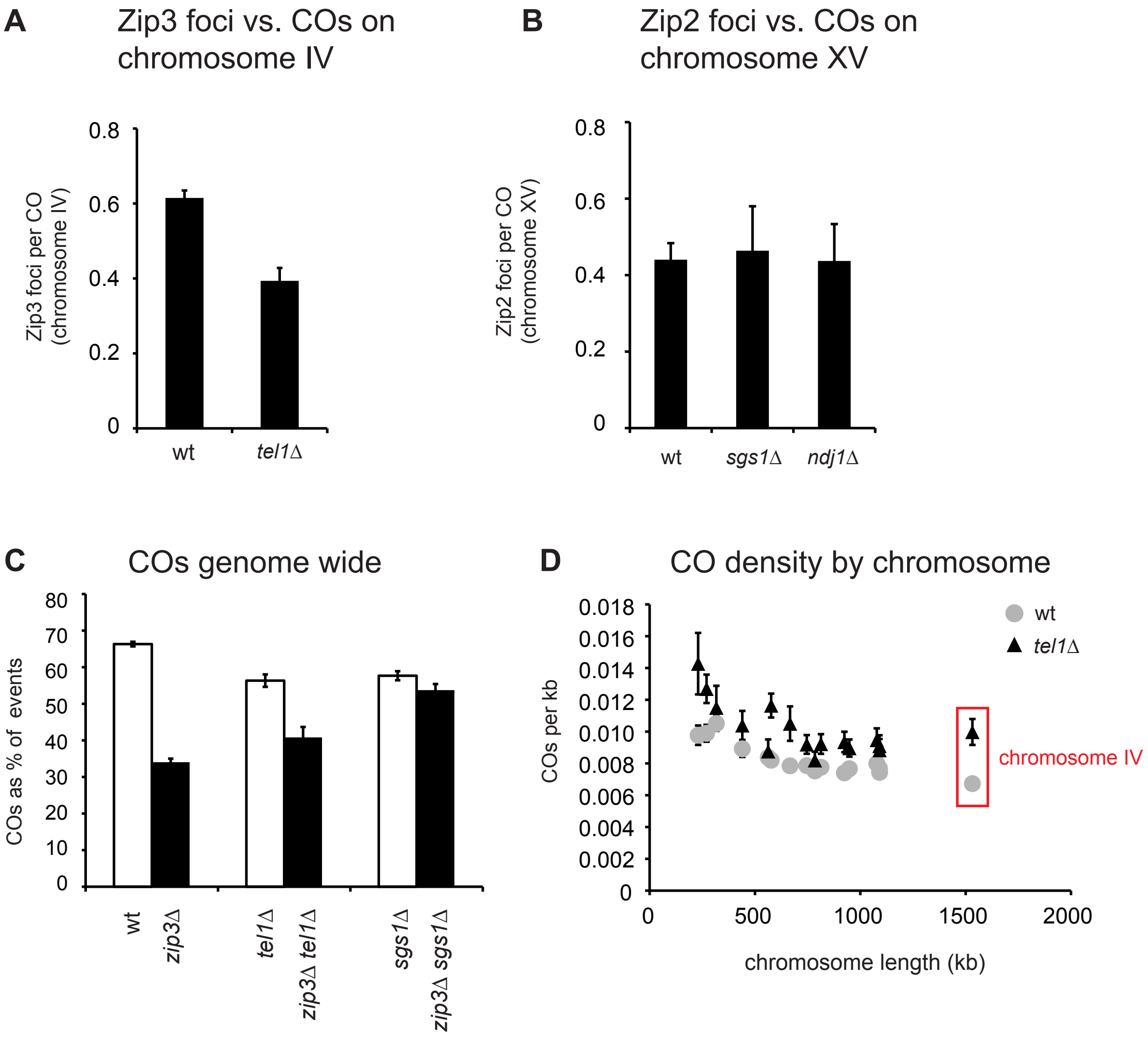

A
Zip3 foci vs. COs on chromosome IV
Zip3 foci per CO (chromosome IV)
0.8
0.6
0.4
0.2
0
wt
tel1Δ
B
Zip2 foci vs. COs on chromosome XV
Zip2 foci per CO (chromosome XV)
0.8
0.6
0.4
0.2
0
wt
sgs1Δ
ndj1Δ
C
COs genome wide
COs as % of events
80
70
60
50
40
30
20
10
0
wt
zip3Δ
tel1Δ
zip3Δ tel1Δ
sgs1Δ
zip3Δ sgs1Δ
D
CO density by chromosome
COs per kb
0.018
0.016
0.014
0.012
0.010
0.008
0.006
0.004
0.002
0
wt
tel1Δ
chromosome IV
0
500
1000
1500
2000
chromosome length (kb)
