# Supplementary material for: Reduced Crossover Interference and Increased ZMM-Independent Recombination in the Absence of Tel1/ATM
Source: PLoS Genet. 2015 Aug 25;11(8):e1005478. doi: 10.1371/journal.pgen.1005478 (PMC4549261; doi:10.1371/journal.pgen.1005478)
Supplement: S1 Table — (PDF) [file pgen.1005478.s009.pdf]

Supporting Table S1: Yeast strains

Most experiments were performed with multiple isolates of a given genotype, as listed below.
The genotype of all BR1919 strains is *leu2-3,122 his4-260 ura3-1 ade2-1 thr1-4 lys2 trp1-289*, plus additional features listed below. BR1919 diploids are fully homozygous at all loci except *MAT*.
S96 and YJM789 haploid parents used to generate hybrids for recombination mapping are listed below.
The genotype of all S96 strains is *MATa ho lys5*, plus additional features as listed.
The genotype of all YJM789 strains is *MATα ho::hisG lys2 cyh*, plus additional features as listed.

Strain names	Strain number(s)	Strain background	Relevant genotype
wt S96	JCF1100	S96	
wt YJM789	JCF4411	YJM789	
tel1Δ S96	yCA694, 695	S96	*tel1Δ::KAN*
tel1Δ YJM789	yCA703, 704	YJM789	*tel1Δ::KAN*
sgs1Δ S96	SYC1120, yCA1318, 1319, 1320	S96	*sgs1Δ::KAN* (SYC1120), *sgs1Δ::NAT* (yCA1318, 1319, 1320)
sgs1Δ YJM789	SYC1121, YCA1321, 1322, 1323	YJM789	*sgs1Δ::KAN* (SYC1121), *sgs1Δ::NAT* (yCA1321, 1322, 1323)
zip3Δ S96	yCA223, 224, 225	S96	*zip3Δ::KAN*
zip3Δ YJM789	yCA291, 292, 293	YJM789	*zip3Δ::KAN*
msh4Δ S96	SYC1110	S96	*msh4Δ::KAN*
msh4Δ YJM789	SYC1111	YJM789	*msh4Δ::KAN*
zip3Δ tel1Δ S96	yCA1126, 1127	S96	*zip3Δ::KAN tel1Δ::NAT*
zip3Δ tel1Δ YJM789	yCA1085, 1086	YJM789	*zip3Δ::KAN tel1Δ::NAT*
zip3Δ sgs1Δ S96	yCA1130, 1131	S96	*zip3Δ::KAN sgs1Δ::NAT*
zip3Δ sgs1Δ YJM789	yCA1083, 1084	YJM789	*zip3Δ::KAN sgs1Δ::NAT*
wt (chr IV labeled at position 1242816)	yCA1442, 1444	BR1919	*MATa/α leu2::tetR-mCherry-HYG/leu2::tetR-mCherry-HYG chrIV-R::tetOarray-URA3/chrIV-R::tetOarray-URA3 Zip3-GFP::KAN/Zip3-GFP::KAN*
tel1Δ (chr IV labeled at position 1242816)	yCA1443, 1445	BR1919	*MATa/α leu2::tetR-mCherry-HYG/leu2::tetR-mCherry-HYG chrIV-R::tetOarray-URA3/chrIV-R::tetOarray-URA3 Zip3-GFP::KAN/Zip3-GFP::KAN tel1Δ::NAT/tel1Δ::NAT*
wt (chr XIV labeled at position 743894)	yCA1076	BR1919	*MATa/α leu2::tetR-mCherry-HYG/leu2::tetR-mCherry-HYG chrXIV-R::tetOarray-THR1/chrXIV-R::tetOarray-THR1 Zip3-GFP::KAN/Zip3-GFP::KAN*
tel1Δ (chr XIV labeled at position 743894)	yCA1077	BR1919	*MATa/α leu2::tetR-mCherry-HYG/leu2::tetR-mCherry-HYG chrXIV-R::tetOarray-THR1/chrXIV-R::tetOarray-THR1 Zip3-GFP::KAN/Zip3-GFP::KAN tel1Δ::NAT/tel1Δ::NAT*

Strain names	Strain number(s)	Strain background	Relevant genotype
zip1Δ	yCA1264	BR1919	*MATa/α zip1Δ::LYS2/zip1Δ::LYS2*
zip1Δ sgs1Δ	yCA1260	BR1919	*MATa/α zip1Δ::LYS2/zip1Δ::LYS2 sgs1Δ::KAN/sgs1Δ::KAN*
zip1Δ tel1Δ	yCA1262	BR1919	*MATa/α zip1Δ::LYS2/zip1Δ::LYS2 tel1Δ::NAT/ tel1Δ::NAT*
