# Supplementary material for: Reduced Crossover Interference and Increased ZMM-Independent Recombination in the Absence of Tel1/ATM
Source: PLoS Genet. 2015 Aug 25;11(8):e1005478. doi: 10.1371/journal.pgen.1005478 (PMC4549261; doi:10.1371/journal.pgen.1005478)
Supplement: S2 Table — (PDF) [file pgen.1005478.s010.pdf]

Supporting Table S2: Tetrads genotyped

Data for *tel1*Δ*, zip3*Δ *tel1*Δ, and two out of six *zip3*Δ *sgs1*Δ tetrads have been deposited in NIH Sequence Read Archive under accession number SRP044001. Data for wild type, *sgs1*Δ, *zip3*Δ, *msh4*Δ*,* and four out of six *sgs1*Δ *zip3*Δ tetrads were previously deposited under accession numbers SRP028549 (wild type) and SRP041214 (all other strains).

Genotype	Tetrads sequenced with high coverage	Tetrads sequenced with lower coverage	Tetrads genotyped by microarray	Notes
wildtype	wtx29<br>wtx30<br>wtx46<br>wtx63<br>wtx64<br>wtx65			Data from 46 tetrads genotyped by high-density tiling array by Mancera et al. (2008) were also used.
*tel1*Δ	tel1x6<br>tel1x7<br>tel1x10<br>tel1x11<br>tel1x12<br>tel1x15<br>tel1x16<br>tel1x18	tel1x5<br>tel1x14<br>tel1x17<br>tel1x19	tel1x2<br>tel1x13	
*sgs1*Δ	sgs1x2<br>sgs1x7<br>sgs1x8<br>sgs1x30<br>sgs1x32<br>sgs1x33<br>sgs1x34<br>sgs1x35<br>sgs1x36	sgs1x29<br>sgs1x31	sgs1x3<br>sgs1x9	
*zip3*Δ	zip3x262<br>zip3x265<br>zip3x268<br>zip3x274<br>zip3x276<br>zip3x289<br>zip3x295	zip3x269<br>zip3x271<br>zip3x272	zip3x251, 254, 255, 256, 257, 264, 266, 273, 275, 277, 278, 290, 292, 296, 297, 298, 299, 300, 301, 303	

Genotype	Tetrads sequenced with high coverage	Tetrads sequenced with lower coverage	Tetrads genotyped by microarray	Notes
*msh4*Δ	msh4x1<br>msh4x2<br>msh4x3<br>msh4x4<br>msh4x5<br>msh4x6<br>msh4x8			Data from 5 tetrads genotyped by high-density tiling array by Mancera et al. (2008) were also used.
*zip3*Δ *tel1*Δ	zip3tel1x1<br>zip3tel1x2<br>zip3tel1x3<br>zip3tel1x5<br>zip3tel1x14<br>zip3tel1x19			
*zip3*Δ *sgs1*Δ	zip3sgs1x2<br>zip3sgs1x4<br>zip3sgs1x5<br>zip3sgs1x7<br>zip3sgs1x8<br>zip3sgs1x10			
