# Supplementary material for: Reduced Crossover Interference and Increased ZMM-Independent Recombination in the Absence of Tel1/ATM
Source: PLoS Genet. 2015 Aug 25;11(8):e1005478. doi: 10.1371/journal.pgen.1005478 (PMC4549261; doi:10.1371/journal.pgen.1005478)
Supplement: S1 Text — (PDF) [file pgen.1005478.s011.pdf]

Supporting Materials and Methods

Yeast strain and plasmid construction

The Zip3-GFP tag and all knockouts except *zip1Δ::LYS2* were made by PCR-mediated gene disruption [1]. *zip1Δ::LYS2* was introduced by transformation with plasmid pMB116 [2]. Strains with arrays of tet operators on chromosome IV were made by integration of plasmid pCA022 (previously called BAM145, a gift from Amy MacQueen) at position 1,242,816. Strains with arrays of tet operators on chromosome XIV were made by integration of plasmid pCA029 at position 743894. pCA029 was constructed by replacing a ClaI-SphI fragment in pCA023 (previously called BAM151, a gift from Amy MacQueen) with a PCR product spanning chromosome XIV positions 743592-744297. tetR-mCherry fusions are derived from strain AM2487 (a gift from Amy MacQueen) and were introduced into yCA strains by crossing.

Antibody staining of meiotic chromosome spreads

For Figure 4 and 5C, spreads were stained with rabbit anti-Zip1 antibody (1:100 dilution) and chicken anti-GFP (Abcam, 1:1000 dilution). Secondary antibodies were goat anti-chicken AlexaFluor 488 (Molecular Probes, 1:200 dilution) and donkey anti-rabbit Cy5 (Jackson, 1:200 dilution). mCherry was visualized without antibody detection. DNA was stained with 1.5 μg/ml DAPI. For Figure 5E, spreads were stained with mouse anti-Red1 antibody (1:50 dilution) and donkey anti-mouse Texas Red (Jackson, 1:200 dilution).

Recalculation of T2 products at *HIS4LEU2* genotyped by Zhang et al.

As defined by Zhang et al. [3] T2 includes COs with a discontinuous GC, our "minority event" categories on two, three, and four chromatids, and 4:0 tracts. Zhang et al. excluded one particular class of two-chromatid events (those with overlapping NCOs on two chromatids) from their T2 category for reasons that are unclear. For comparison with our results, we recalculated the number of T2 events at *HIS4LEU2* in wt and *tel1*Δ using their raw data, including overlapping NCOs as T2 events. Recalculated numbers are 20% (wild type) and 36% (*tel1*Δ) of detectable recombination products as stated in the main text.

Supporting References

1. Longtine MS, McKenzie A, 3rd, Demarini DJ, Shah NG, Wach A, et al. (1998) Additional modules for versatile and economical PCR-based gene deletion and modification in *Saccharomyces cerevisiae*. Yeast 14: 953-961.
2. Sym M, Roeder GS (1994) Crossover interference is abolished in the absence of a synaptonemal complex protein. Cell 79: 283-292.
3. Zhang L, Kim KP, Kleckner NE, Storlazzi A (2011) Meiotic double-strand breaks occur once per pair of (sister) chromatids and, via Mec1/ATR and Tel1/ATM, once per quartet of chromatids. Proc Natl Acad Sci U S A 108: 20036-20041.
